# Greater temporo-occipital gray matter volume and frontoparietal cortical thickness in post-menopausal women: a UK Biobank study

**DOI:** 10.1007/s00429-026-03206-5

**Published:** 2026-10-09

**Authors:** Stefanos Tyrovolas, Maria J. Portella, Judith Usall, Maria Rosa Cambra-Martí, Antonio Callén, Josep Maria Haro, Christian Núñez, Christian Stephan-Otto

**Affiliations:** 1 Department of Nutrition and Food Studies, George Mason University, Fairfax, VA 22030 USA; 2 School of Nursing, The Hong Kong Polytechnic University, Hong Kong, Hong Kong SAR, China; 3 Research, Innovation and Teaching Unit, Parc Sanitari Sant Joan de Déu, 08830 Sant Boi de Llobregat, Spain; 4 Centro de Investigación Biomédica en Red de Salud Mental (CIBERSAM), Instituto de Salud Carlos III, 28029 Madrid, Spain; 5 Mental Health Research Group, Institut d’Investigació Biomèdica Sant Pau (IIB Sant Pau), Hospital de La Santa Creu i Sant Pau, Barcelona, Spain; 6 Department of Psychiatry and Forensic Medicine, Universitat Autònoma de Barcelona, Barcelona, Spain; 7 Institut de Recerca Sant Joan de Déu, Esplugues de Llobregat, Barcelona Spain; 8 Radiology Department, Parc Sanitari Sant Joan de Déu, Sant Boi de Llobregat, Barcelona Spain; 9 Neurology Department, Parc Sanitari Sant Joan de Déu, Sant Boi de Llobregat, Barcelona Spain; 10 Fundació de Recerca Clínic Barcelona - Institut d’Investigacions Biomèdiques August Pi I Sunyer (IDIBAPS), Barcelona, Spain; 11 Pediatric Computational Imaging Center (PeCIC), Hospital Sant Joan de Déu, Esplugues de Llobregat, Barcelona Spain

**Keywords:** Menopause, Brain Structure, Neuroimaging

## Abstract

Menopause is a critical transitional stage in women’s lives, yet evidence on its impact on brain structure remains inconsistent, and large-sample neuroimaging studies are limited. Here, we applied whole-brain morphometry techniques to evaluate differences between pre- and post-menopausal women in terms of gray matter volume, cortical thickness and cortical gyrification. We processed and analyzed T1-weighted brain scans of 4098 women (1033 pre-menopausal; 3065 post-menopausal) from the UK Biobank. Post-menopausal women exhibited greater gray matter volume than pre-menopausal women primarily in the right temporal lobe, encompassing the amygdala and the hippocampus, and extending to the left temporal lobe and occipital regions. Post-menopausal women also exhibited greater cortical thickness in the frontal lobe and the supramarginal gyrus, whereas no differences in cortical gyrification were observed. These findings were consistent across multiple sensitivity analyses addressing age differences, peri-menopausal status, and other potential confounds. The spatial pattern of differences between post-menopausal and pre-menopausal women considerably overlaps with regions affected early in Alzheimer’s disease, such as the medial temporal lobe and the supramarginal gyrus. Our findings of larger gray matter volume and greater cortical thickness in post-menopausal women may reflect compensatory mechanisms. Speculatively, these findings align with the biphasic model of brain structural changes in Alzheimer’s disease. Further research with longitudinal designs is required to elucidate the exact mechanisms underlying the brain structural differences associated with menopause.

**Supplementary Information:**

The online version contains supplementary material available at https://doi.org/10.1007/s00429-026-03206-5.

## Introduction

The effect of aging is different in men and women as demonstrated by differences in healthy life expectancy, disability, and disease burden (Guo et al. 2025). While advanced age has been reported as a major factor for brain volume changes, which have been related with several brain-related disorders, female sex is another important determinant (Than et al. 2021). Recent evidence emphasizes that women are more affected by neurological related disorders, like dementias, than men (Guo et al. 2025). For instance, data from the global burden of disease (GBD) study showed that the burden of neurological diseases in women is a serious global health challenge (Guo et al. 2025), however the pathways and factors related to this increased burden in women are still understudied.

Several previous research works have noted that age and sex are related to brain health and alterations in brain volume (Wang et al. 2019) but have not evaluated this in an extended way. Menopause is an important phase in a woman’s life and is associated with a plethora of hormonal changes. It is a natural life-stage transition reflecting the end of reproductive capability (Santoro et al. 2021). Given the neuroprotective effects of estradiol (McEwen et al. 2012) and the observed cognitive decline associated with peri-menopause—where some longitudinal studies show a recovery in post-menopause (Greendale et al. 2009) while others report persistent deficits (Epperson et al. 2013; Maki et al. 2021)—the study of how menopausal status is associated with brain structure is particularly relevant. A recent review focused on brain differences in menopause and their association with cognitive performance, mostly including cross-sectional studies, has highlighted differences in brain volume across menopausal stages (Ramli et al. 2023). In parallel, another review on brain anatomy, menopause, and the effects of menopausal hormone therapy found that most studies suggest at least some beneficial effects of estrogens on the brain (defined as the observation of more brain tissue in pre-menopausal women or post-menopausal women using menopausal hormone therapy), although the evidence is mixed (Luders et al. 2025). It is important to note, however, that structural differences may reflect not only hormonal fluctuations, but also the emergence of menopausal symptoms that can consequently impact brain health (Maki and Thurston 2020).

Most studies in this field have reported smaller volumes in post-menopausal women compared with pre- or peri-menopausal women (Than et al. 2021; Ramli et al. 2023; Zuhlsdorff et al. 2026). Smaller brain volumes in post-menopause have been associated with poorer cognition, reduced blood flow, and reduced glucose metabolism, among others (Ramli et al. 2023). However, other reports suggest that brain volume dynamics are complex, sometimes presenting a larger brain volume linearly associated with time elapsed since menopause (Ambikairajah et al. 2021), and sometimes showing gray matter volume increases in a group of post-menopausal women after a 2-year follow-up, which could potentially be associated with compensatory mechanisms specific to this stage (Mosconi et al. 2021). Until today, however, there are limited big data studies with neuroimaging (using the UK Biobank dataset) that have analyzed potential associations between menopausal status and brain structure (Ambikairajah et al. 2021; Than et al. 2021; Zuhlsdorff et al. 2026) (see a summary of these studies in Table 1). In addition, these studies either analyzed global brain measures or were limited to a few predefined regions of interest, thereby lacking anatomical specificity and/or failing to provide a comprehensive picture of how menopause shapes the structure of the brain.

**Table 1:** Overview of UK Biobank neuroimaging studies investigating menopause-related brain structural differences

Studies	Sample	Main findings
(Than et al. 2021)	N = 2,057 women (230 pre/peri-menopausal women; 1,827 post-menopausal women). Neuroimaging analyses based on UK Biobank image-derived phenotypes	Menopause interacted with age in predicting brain structure. Post-menopausal women showed a steeper age-related decline in total brain volume and gray matter volume and also had a greater white matter hyperintensity burden than pre/peri-menopausal women
(Ambikairajah et al. 2021)	N = 5,072 women aged 45–79 years (735 pre-menopausal; 4,337 post-menopausal). Analyzed total brain and hippocampal volumes after excluding HRT users, hysterectomy/oophorectomy, stroke and peri-menopause	Post-menopausal women had larger total brain and hippocampal volumes than pre-menopausal women, including in age-matched analyses. Findings note menopause-related brain changes beyond normal ageing effects
(Mukora et al. 2025)	N = 14,428 women included in the female-specific analyses, and 890 women included in the analysis of menopause subgroup (222 pre-menopausal; 445 natural menopause; 223 surgical menopause)	Reports menopause-dependent relationships between latent patterns of modifiable dementia risk factors and cortical thickness, although strict age matching attenuated these findings
(Zuhlsdorff et al. 2026)	N = 10,873 women included. in the neuroimaging analysis (762 pre-menopausal; 7,583 post-menopausal with no HRT history; and 2,528 post-menopausal with HRT history). Analyses focused on mental health, cognition, and selected brain regions including the medial temporal lobe and anterior cingulate cortex	Menopause was associated with smaller medial temporal lobe and anterior cingulate cortex volumes and poorer mental health outcomes
Present study	N = 4,098 women (1,033 pre-menopausal; 3,065 post-menopausal women), aged ≤58 years from the UK Biobank database. Whole-brain voxel-based and surface-based morphometry analyses were applied	This study performed whole-brain voxel-wise analyses of gray matter volume, cortical thickness, and cortical gyrification. Post-menopausal women exhibited greater temporo-occipital gray matter volume and greater frontoparietal cortical thickness, with no gyrification differences

To provide a comprehensive evaluation of brain structure in relation to menopause, here we assessed three distinct but complementary brain morphometric parameters. Gray matter volume is a macroscopic composite measure that reflects a combination of cortical thickness and surface area, albeit more closely related to the latter (Winkler et al. 2010). Brain surface parameters, such as cortical thickness (the distance between the white matter surface and the pial surface) and cortical gyrification (representing the absolute mean curvature of the surface), may capture complementary information and provide interesting insights into brain cytoarchitecture (Fischl et al. 2008; Winkler et al. 2010; Ronan and Fletcher 2015) and connectivity (Hogstrom et al. 2013).

Taking into account all the aforementioned, the present study aims to evaluate differences between independent groups of pre- and postmenopausal women in brain structure using data from the UK Biobank, which is among the few large-scale biomedical databases in the world that contain neuroimaging data and information on menopausal status. We employed whole-brain morphometry techniques that allow us to precisely and unbiasedly localize brain structure differences associated with menopause. Aside from gray matter volume differences, we studied—for the first time using whole-brain morphometry techniques, to the best of our knowledge—the potential association between menopausal status and both cortical thickness and cortical gyrification. Given that existing evidence is inconclusive, along with the lack of studies examining cortical thickness and gyrification in the context of menopause, this study is fundamentally exploratory. As aforementioned, evidence is emerging showing that the burden of neurological disorders and disability is increasing at alarming rates among women; hence, the information derived from our study may provide important pointers for effective targeted public health planning.

## Methods

### Participants and menopause data

**Fig. 1:**
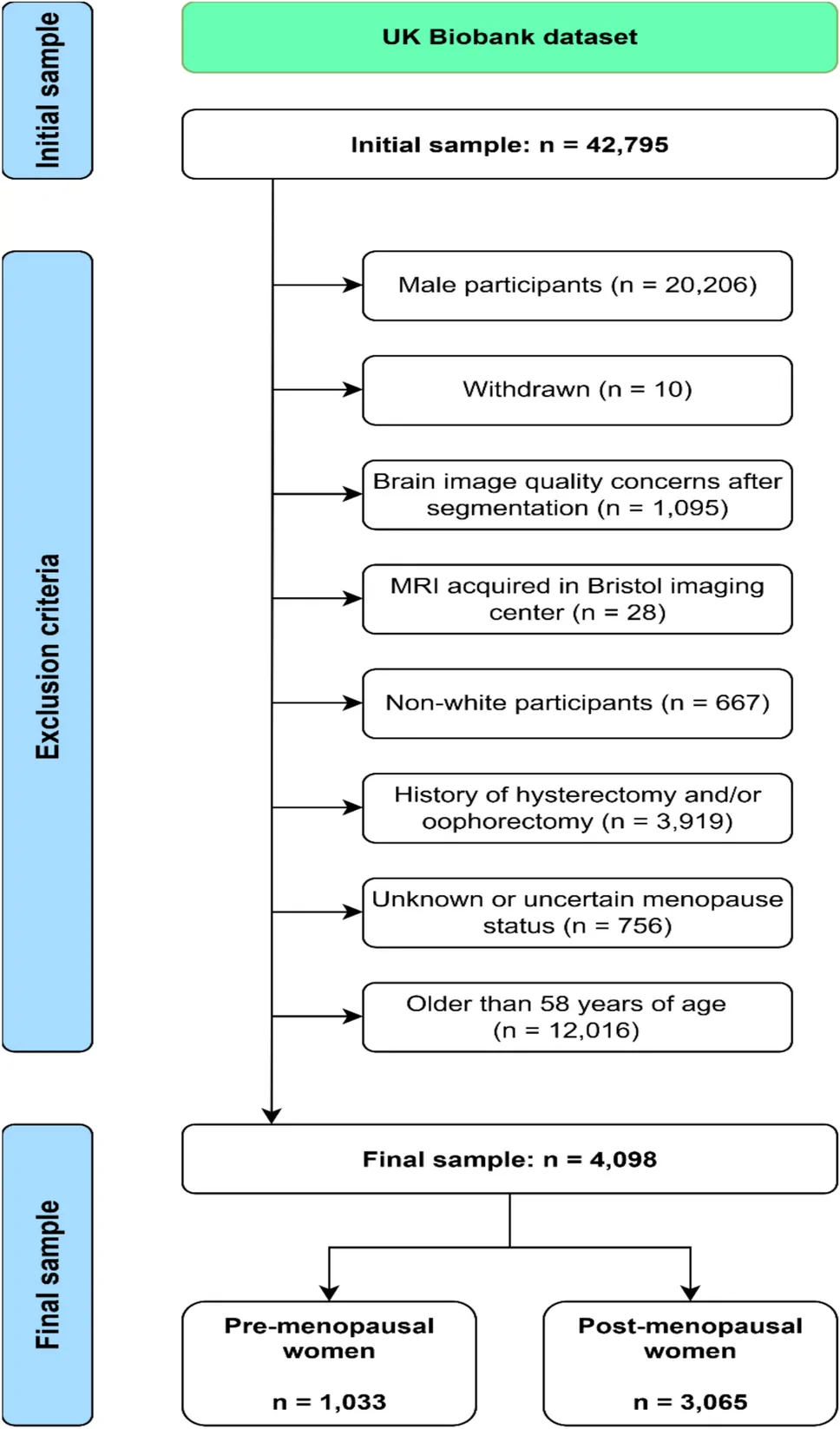
Study flow diagram. Overview of the exclusion criteria applied for participant selection from the UK Biobank dataset. The final sample included 4098 women, stratified into pre-menopausal (n = 1033) and post-menopausal (n = 3065) groups

Participants were gathered from the UK Biobank dataset (project ID: 72377). UK Biobank (Hewitt et al. 2016) is a large-scale biomedical database and research resource containing genetic, lifestyle, and health information from half a million UK participants. From an initial sample of 42,795 that had a structural brain image available, we excluded males (n = 20,206).

and participants who decided to withdraw from the study (n = 10). We also dismissed some female participants for concerns about brain image quality after performing the segmentation procedure (n = 1095), as well as those that had been acquired in the Bristol imaging center, since they represented an extremely low proportion of participants (0.1%, n = 28). Similarly, given that non-white participants constituted a very small and highly heterogeneous group, we excluded participants who reported a different ethnic background from white or who did not disclose their ethnicity (3.1%, n = 667). We further excluded women who reported having undergone a hysterectomy and/or an oophorectomy (or did not answer/were not sure; n = 3919). We collected information on menopausal status at the moment of the brain scan (data-field 2724–2.0). Participants were specifically asked the following question: “Have you had your menopause (periods stopped)?”. We excluded women who did not answer or were not sure (n = 756). Finally, to avoid potentially confounding effects due to age, we disregarded women older than 58 (n = 12,016), as this is considered the natural upper age limit for menopause (Shifren et al. 2014). A flow diagram summarizing these exclusion criteria is available in Fig. 1. After all these exclusions, we analyzed a final sample of 4098 women, 1033 pre-menopausal (mean age = 51.5 ± 2.3 [range: 45.2–57.7]), and 3065 post-menopausal (mean age = 55.0 ± 2.2 [range: 47.1–58.0]). All participants were confirmed as biological females based on the genetic sex variable provided by the UK Biobank (data-field 22,001–0.0). It should be noted that the UK Biobank recruited exclusively middle-aged to older adults; therefore, the lower age limit of our sample was naturally restricted by the cohort.

### Neuroimaging data acquisition and processing

Brain scans were acquired between May 2014 and March 2020 at one of three imaging centers (Cheadle, Reading, and Newcastle) arranged by UK Biobank. The full imaging protocol is available here: https://biobank.ndph.ox.ac.uk/ukb/ukb/docs/brain_mri.pdf. We processed all.

T_1_-weighted structural brain images with CAT (v12.7, r1742) (Gaser et al. 2024), a toolbox for SPM12 (https://www.fil.ion.ucl.ac.uk/spm/software/spm12), running under MATLAB (Release 2020b, The MathWorks, Inc., Natick, MA, USA). Image processing involves brain tissue segmentation (gray matter, white matter, and cerebrospinal fluid) and the reconstruction of the cortical surface. This procedure allowed us to generate precise brain maps of gray matter volume, cortical thickness, and cortical gyrification. Gyrification values were estimated with the absolute mean curvature method (Luders et al. 2006). Gray matter volume maps were smoothed with an 8 mm full width at half maximum (FWHM) filter. Cortical thickness and cortical gyrification maps were resampled to a high-resolution 164 k mesh and smoothed with 12 mm and 20 mm FWHM filters, respectively, following CAT developers’ recommendations. We used these processed gray matter volume and surface (cortical thickness and cortical gyrification) maps to perform voxel-based morphometry (VBM) and surface-based morphometry (SBM) analyses. To ensure the good quality of the data included in the analyses, we used the “Data quality” tools provided in CAT and the segmentation procedure reports to identify participants who might have been processed inadequately. As noted earlier, we excluded 1095 (4.8% of all females) participants after this quality check.

### Statistical analysis

To analyze potential brain differences between pre-menopausal and post-menopausal women, we carried out whole-brain ANCOVA VBM and SBM analysis of the gray matter volume, cortical thickness, and cortical gyrification brain maps. The confound modeling was based on the recommendations by Alfaro-Almagro et al. 2021, as we adopted their “simple” set of confounds. Thus, the following covariates were included: age, age squared, total intracranial volume, head motion in resting fMRI, head motion in task fMRI, date (days elapsed since the start of acquisitions), and date squared. Given that acquisition site is a strong confounding factor in multi-site studies, each of these covariates was represented three times (one per site) to account for site-by-covariate interactions, while the main effect of site itself was modeled using two dummy variables (Alfaro-Almagro et al. 2021), resulting in a total of 23 covariates. Of note, we used functional head motion as a proxy for structural motion, as in-scanner movement has been shown to be a highly stable trait within individuals (Savalia et al. 2017). Moreover, the inclusion of the number of days since the start of acquisitions accounts for scanner drift and other long-term temporal effects across the data collection period. Bonferroni correction was applied to account for the testing of three brain parameters. Since age differed significantly between groups, we aimed to minimize this difference by conducting an additional ANCOVA analysis, which included only the youngest post-menopausal women to match the number of pre-menopausal participants (n = 1033 each group). We did not use exact age-matching algorithms to preserve statistical power and cohort representativeness. Moreover, to address potential confounding factors, we repeated the original analysis by including additional covariates, one at a time, or excluding participants who met a given criterion. All the additional covariates and exclusion criteria that were tested are detailed in Supplementary Table 1. These covariates were included in our sensitivity analysis to account for their potential impact on brain integrity. For instance, body mass index, physical activity, and sleep-related symptoms have been associated with structural brain alterations in previous UK Biobank neuroimaging studies (Hamer and Batty 2019; Yu et al. 2023; Hoogen et al. 2025). To further contextualize our findings and isolate the impact of age on brain structure from that associated with menopause, we conducted whole-brain linear regression analyses in males, using age as a predictor and the aforementioned covariates (except age and age squared). Finally, to refine the biological interpretation of our results, we conducted post-hoc analyses. Specifically, we separated women from the original pre-menopausal group who reported an irregular menstrual cycle or a cycle length below 24 days or above 38 days (data-field 3710–2.0). This allowed us to form a subgroup of women likely undergoing peri-menopause (Harlow 2018). To avoid bias, women who did not report their menstrual cycle length were excluded from these analyses. We then compared brain differences between the newly defined pre- and peri-menopausal groups, as well as between each of these and the post-menopausal group. For both VBM and SBM analyses, we used a cluster-forming threshold of p < 0.001, uncorrected at the voxel or vertex level, respectively. Statistical significance was subsequently determined by family-wise error (FWE) correction applied at the cluster-extent level. The minimum cluster size was defined as the extent required to survive the Bonferroni-adjusted cluster-level threshold of FWE p < 0.016. Identical thresholds were used for both VBM and SBM analyses. Furthermore, a standard gray matter masking threshold of 0.2, which is within the range recommended by CAT developers, was applied in gray matter volume analysis to restrict the results to voxels with a gray matter probability greater than or equal to 0.2, to ensure that non-brain areas were not included. We used Neuromorphometrics (https://neuromorphometrics.com) and Desikan-Killiany (DK40) (Desikan et al. 2006) atlases to precisely localize the results from our VBM and SBM analyses, respectively. For interpretability purposes, we also used the Neurosynth Image Decoder tool (https://neurosynth.org/decode; accessed on February 11, 2026) (Yarkoni et al. 2011) to identify functional and cognitive terms spatially correlated with our VBM results.

## Results

### Sociodemographic, reproductive, and clinical variables compared between groups

As expected, post-menopausal women were significantly older than pre-menopausal women (p < 0.001, Cohen’s d = 1.57, overlap coefficient = 45.7%). Body mass index (p = 0.038) and sleep duration (p < 0.001) were higher in the pre-menopausal group, while physical activity (p = 0.031) and the percentage of women who used menopausal hormone therapy (referred to as hormone-replacement therapy in the UK Biobank; p < 0.001) were higher in post-menopausal women. Other variables, such as menarche age, use of oral contraceptives, and number of pregnancies, were not different between groups (Table 2). The comparison of the pre-menopausal group with the youngest post-menopausal women yielded similar results (Supplementary Table 2).

**Table 2:** Comparison of sociodemographic, reproductive, and clinical variables between pre-menopausal and post-menopausal women. Values are expressed as mean ± SD for continuous variables or percentages for categorical variables

Variable	Pre-menopausal women (n = 1,033)	Post-menopausal women (n = 3,065)	Difference^e^
Age (years)	51.5 ± 2.3	55.0 ± 2.2	**p < 0.001**
Menarche age (years)	13.2 ± 1.6	13.1 ± 1.5	p = 0.397
Ever used oral contraceptives (% yes)	88.3	89.9	p = 0.142
Age started oral contraceptives	19.3 ± 4.3	19.3 ± 3.8	p = 0.964
Years using oral contraceptives	12.0 ± 9.7	11.8 ± 9.5	p = 0.665
Number of live births	1.5 ± 1.2	1.5 ± 1.2	p = 0.690
Number of total pregnancies	2.0 ± 1.6	2.0 ± 1.6	p = 0.851
Body mass index	26.2 ± 5.0	25.8 ± 4.8	**p = 0.023**
Sleep duration (h)	7.2 ± 1.0	7.0 ± 1.0	**p < 0.001**
Physical activity (MET-min/week)	2,536 ± 2,270	2,714 ± 2,381	**p = 0.047**
Current tobacco use (%)^a^	96 / 2 / 2	96 / 2 / 2	p = 0.727
Past tobacco use (%)^b^	53 / 18 / 14 / 14	53 / 18 / 13 / 17	p = 0.325
Current alcohol use (%)^c^	5 / 11 / 17 / 31 / 24 / 11	6 / 12 / 16 / 30 / 26 / 11	p = 0.816
Townsend deprivation index	-1.5 ± 2.8	-1.6 ± 2.8	p = 0.338
Experiences following childhood^d^	2 (4)	2 (4)	p = 0.740
Serious life events^d^	0 (1)	0 (1)	p = 0.114
Ever depressed for a whole week (% yes)	59.4	61.3	p = 0.273
Diagnosed with any mental illness (% yes)	3.6	4.0	p = 0.566
Menopausal hormone therapy (% yes)	8.0	22.7	**p < 0.001**

^a^Categories for current tobacco use: 0 = No; 1 = Only occasionally; 2 = Yes, on most or all days

^b^Categories for past tobacco use: 0 = I have never smoked; 1 = Just tried once or twice; 2 = Smoked occasionally; 3 = Smoked on most or all days. Note that percentages do not add up to 100% due to rounding

^c^Categories for current alcohol use: 0 = Never; 1 = Special occasions only; 2 = One to three times a month; 3 = Once or twice a week; 4 = Three or four times a week; 5 = Daily or almost daily. Note that percentages do not add up to 100% due to rounding

^d^Reported as median (interquartile range)

^e^p values are derived from t-tests for continuous variables and chi-squared tests for categorical variables

MET = Metabolic Equivalent Task

### Main analysis

**Fig. 2:**
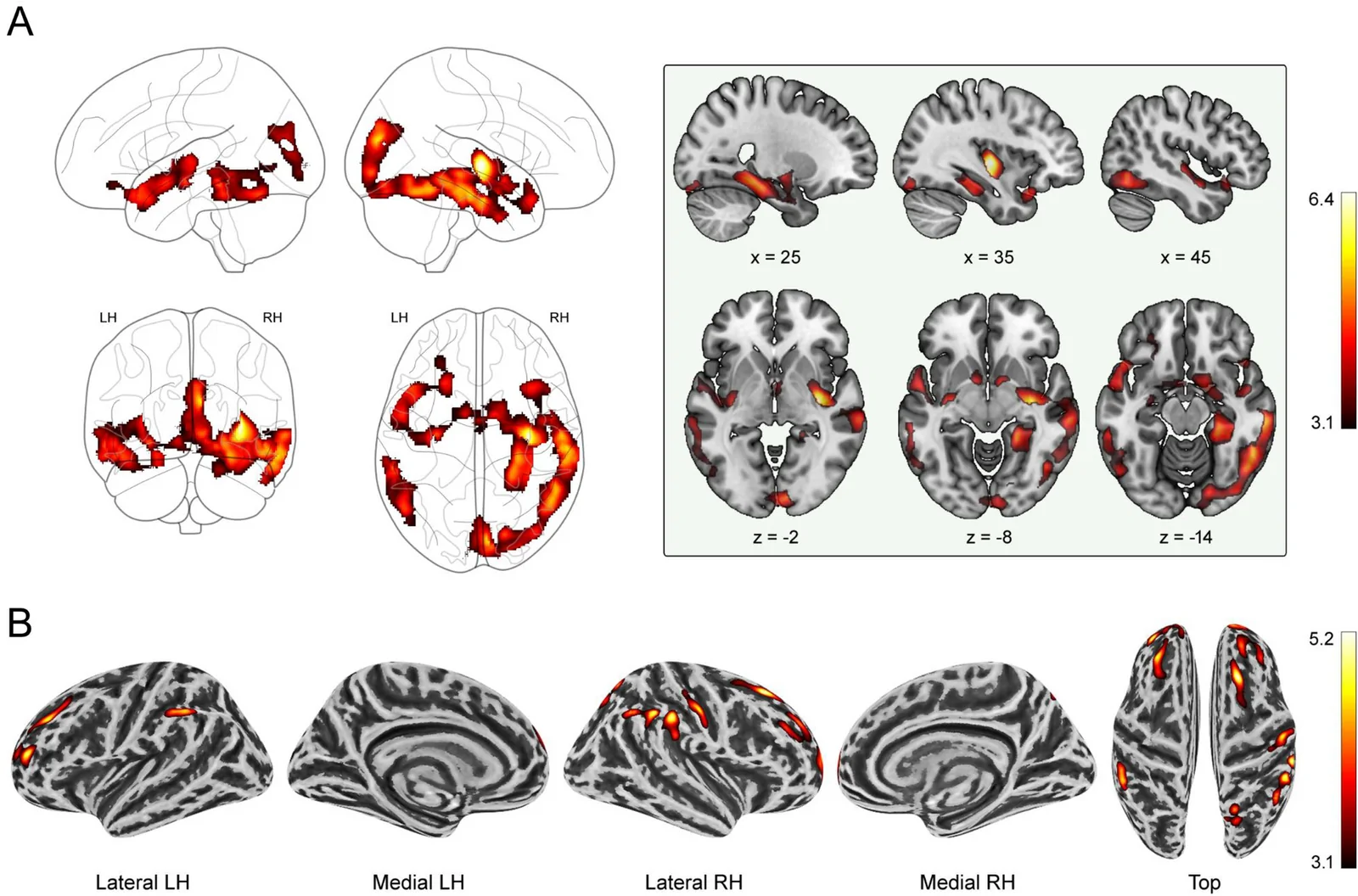
Results of the whole-brain morphometry analyses. Brain clusters where post-menopausal women (n = 3065) showed greater gray matter volume (**A**) or cortical thickness (**B**) than pre-menopausal women (n = 1033). This analysis was controlled for the 23 covariates described in the main text. Only clusters that survived Bonferroni correction, accounting for the three brain parameters analyzed, are shown (p < 0.0167). Color bars represent T values and the numbers below the axial slices indicate the “z” MNI coordinate. See Tables 3 and 4 for a list of the brain regions depicted in this figure

Our analysis comparing pre-menopausal and post-menopausal women revealed two clusters of greater gray matter volume in post-menopausal women (Table 3 and Fig. 2A). The largest cluster (cluster size = 16,533 voxels; p_cluster_ = 3.6×10^–13^; T_peak_ = 6.4; Cohen’s d_peak_ = 0.23; Montreal Neurological Institute (MNI) coordinates = 35, − 15, 2) encompassed the right temporal lobe and part of the right occipital lobe. Regions in the right hemisphere showing a higher percentage of their volume covered by this cluster included the amygdala, the parahippocampal gyrus, the hippocampus, and the cuneus, among others. The second cluster (cluster size = 2,116 voxels; p_cluster_ = 0.002; T_peak_ = 4.8; Cohen’s d_peak_ = 0.17; MNI coordinates = − 59, − 35, − 12) was mainly located in the left temporal lobe, also covering part of the left occipital lobe. Both clusters were still significant after Bonferroni correction. Similarly, the analysis of cortical thickness maps showed several clusters (cluster sizes ranging from 974 to 1,332 vertices) of greater cortical thickness in post-menopausal women compared to pre-menopausal women (Table 4 and Fig. 2B). All the clusters were located either in the frontal or the parietal (mainly the supramarginal gyrus) lobes, revealing no spatial overlap with the gray matter volume findings. We did not find any region showing greater gray matter volumes or cortical thickness in pre-menopausal women. No cortical gyrification differences between groups were found. For completeness, omitting the Bonferroni correction across modalities revealed only two additional clusters of greater cortical thickness in post-menopausal women (p = 0.017, mainly located in the right fusiform gyrus, and p = 0.040, mainly located in the inferior parietal lobule), but no regions showing larger values (gray matter volume, cortical thickness, or cortical gyrification) in the pre-menopausal group.

**Table 3:** Brain regions showing greater gray matter volume in post-menopausal women (n = 3065) compared to pre-menopausal women (n = 1033), controlling for the 23 covariates described in the main text. Only the clusters that survived Bonferroni correction, accounting for the three brain parameters analyzed (p < 0.0167), are listed. For each cluster, we list the main regions in two columns: sorted by the % of total cluster volume localized within that region (% of cluster overlap) and sorted by the % of the total volume of the region covered by the cluster (% of region involvement). See Fig. 2A for a graphical representation of these differences

Cluster size (p value)	Peak t value (Cohen’s d)	MNI coordinates	% of cluster overlap	% of region involvement
16,533 (3.6×10^–13^)	6.4 (0.23)	35, − 15, 2	Right middle temporal gyrus (11%)	Right amygdala (64%)
			Right inferior temporal gyrus (8%)	Right basal forebrain (47%)
			Right lingual gyrus (5%)	Right posterior insula (37%)
			Left temporal pole (4%)	Right parahippocampal gyrus (36%)
			Right cuneus (4%)	Right cuneus (34%)
			Left cuneus (3%)	Right hippocampus (33%)
			Right fusiform gyrus (3%)	Right occipital fusiform gyrus (30%)
			Right hippocampus (3%)	Left accumbens (29%)
			Right occipital fusiform gyrus (3%)	Left planum polare (28%)
			Right parahippocampal gyrus (2%)	Right planum polare (28%)
2,116 (0.002)	4.8 (0.17)	-59, − 35, − 12	Left inferior temporal gyrus (43%)	Left inferior temporal gyrus (18%)
			Left middle temporal gyrus (40%)	Left middle temporal gyrus (13%)
			Left inferior occipital gyrus (5%)	Left inferior occipital gyrus (4%)
			Left superior temporal gyrus (2%)	Left superior temporal gyrus (2%)
			Left fusiform gyrus (2%)	Left fusiform gyrus (1%)
			Left occipital fusiform gyrus (1%)	Left occipital fusiform gyrus (1%)

**Table 4:** Brain regions showing greater cortical thickness in post-menopausal women (n = 3,065) compared to pre-menopausal women (n = 1033), controlling for the 23 covariates described in the main text. Only the clusters that survived Bonferroni correction, accounting for the three brain parameters analyzed (p < 0.0167), are listed. See Fig. 2B for a graphical representation of these differences

Cluster size (p value)	Peak t value (Cohen’s d)	MNI coordinates	Regions in cluster (%)
1,332 (4.9×10^–6^)	5.0 (0.18)	-29, 54, 13	Left rostral middle frontal (62%)
			Left superior frontal (38%)
1,292 (7.4×10^–6^)	4.3 (0.15)	25, 49, 30	Right rostral middle frontal (90%)
			Right superior frontal (10%)
1,278 (8.5×10^–6^)	5.0 (0.18)	48, − 40, 54	Right inferior parietal (50%)
			Right supramarginal (50%)
1,264 (9.8×10^–6^)	4.7 (0.17)	50, − 11, 40	Right postcentral (100%)
1,166 (2.6×10^–5^)	5.1 (0.18)	61, − 27, 42	Right supramarginal (100%)
1,159 (2.8×10^–5^)	4.6 (0.17)	-54, − 38, 49	Left supramarginal (100%)
1,068 (6.9×10^–5^)	4.5 (0.16)	18, 65, 8	Right rostral middle frontal (62%)
			Right superior frontal (32%)
			Right frontal pole (7%)
998 (1.4×10^–4^)	5.2 (0.19)	23, 25, 51	Right superior frontal (100%)
982 (1.6×10^–4^)	4.4 (0.16)	19, − 63, 61	Right superior parietal (98%)
			Right precuneus (2%)
974 (1.8×10^–4^)	4.8 (0.17)	-30, 40, 33	Left rostral middle frontal (71%)
			Left caudal middle frontal (16%)
			Left superior frontal (13%)

### Sensitivity analyses

Selecting only the youngest post-menopausal women reduced the age difference between groups, though it remained significant (pre-menopausal: 51.5 ± 2.3 years old; post-menopausal: 52.4 ± 1.4 years old; p < 0.001, Cohen’s d = 0.49, overlap coefficient = 59.2%). The age distributions for both the full sample and this age-restricted subsample are depicted in Supplementary Fig. 1. The original pattern of results was mostly preserved when we compared pre-menopausal women with the youngest post-menopausal women, albeit the largest cluster in the gray matter volume analysis now included the cerebellum (Supplementary Tables 3 and 4; Supplementary Fig. 2). Similarly, the spatial distribution and magnitude of the effects remained highly stable across all other sensitivity models. No single covariate (such as menarche age, use of oral contraceptives, body mass index, and adverse life events, among others; see Supplementary Table 1) meaningfully attenuated the observed group differences when included in the models. Likewise, excluding women with a diagnosis of mental illness or those who had used menopausal hormone therapy preserved the original spatial patterns, with effect sizes remaining virtually identical to those of the main analysis. For reference, across all sensitivity models, maximum peak effect sizes ranged from a Cohen’s d of 0.21 to 0.25 for gray matter volume, and a Cohen’s d of 0.18 to 0.20 for cortical thickness, compared to the maximum Cohen’s d of 0.23 and 0.19 in our original gray matter volume and cortical thickness analyses, respectively. A graphical depiction of the results for all sensitivity analyses is provided in Supplementary Fig. 3.

### Age effects on brain structure in males

To evaluate the impact of age on brain structure in this age range, independent of menopausal status, we performed whole-brain linear regression analyses of gray matter volume and cortical thickness in males, using age as a predictor. Using exclusion criteria similar to those described previously, we selected a total of 3704 male participants in the age range of our female sample (mean age = 53.9 ± 2.6 [range: 45.4–58.0]). In males, no brain regions showed a positive association between age and gray matter volume or cortical thickness; older age was associated exclusively with lower gray matter volume and cortical thickness throughout the brain (Supplementary Fig. 4). The maximum Cohen’s d_peak_ for the gray matter volume and cortical thickness were 0.27 and 0.28, respectively.

### Characterization of a peri-menopausal subgroup

Based on menstrual cycle length data, we identified a subgroup of women likely undergoing peri-menopause among those labeled as pre-menopausal. Maps of gray matter volume and cortical thickness were compared between the now smaller pre-menopausal group (n = 543) and the newly created peri-menopausal group (n = 431), but no significant differences arose. Furthermore, we observed a similar pattern of gray matter volume and cortical thickness differences when we repeated our original analysis by comparing pre- (without peri-) menopausal women with post-menopausal women (Supplementary Tables 5 and 6; Supplementary Fig. 5). The post-menopausal group showed larger gray matter volumes mainly in temporo-occipital regions, as well as greater cortical thickness in the left supramarginal and frontal areas. Of note, we also found clusters of larger gray matter volume in the cerebellum and greater cortical thickness in the right supramarginal and inferior parietal, which were nominally significant (p < 0.05) but did not pass Bonferroni correction. Finally, brain differences between post- and peri-menopausal women overlapped only partially with those observed in the post- vs. pre-menopausal comparison. Post-menopausal women displayed larger gray matter volume than peri-menopausal women in right temporo-occipital regions, as well as greater cortical thickness in left frontal regions (Supplementary Tables 7 and 8).

### Neurosynth terms correlated with VBM results

We uploaded the unthresholded T-map corresponding to the contrast of interest from our original VBM analysis (showing larger gray matter volumes in the post-menopausal group) to the Neurosynth Image Decoder tool (https://neurosynth.org/decode). Consistent with our anatomical findings, the terms showing the highest correlations were related to memory (e.g., “episodic”, “encoding”, “memories”, “autobiographical”) and emotions (e.g., “emotional”, “neutral”, “expressions”, “fearful”). The top 100 terms identified by Neurosynth are listed in Supplementary Table 9. We observed a highly consistent pattern of terms when we uploaded the unthresholded T-maps from the other analyses (i.e., pre-menopausal vs. youngest post-menopausal, and pre-menopausal [excluding peri-menopausal] vs. post-menopausal).

## Discussion

The present work shows that post-menopausal women presented both larger gray matter volumes and greater cortical thickness when compared with pre-menopausal women, independent of age. Gray matter volume findings included broad regions of the temporal and occipital lobes, predominantly in the right hemisphere, while cortical thickness differences were mainly located in the frontal lobe and the supramarginal gyrus. Importantly, given the relatively high mean age of our pre-menopausal group, we must consider the possibility that our main contrast reflects the difference between the peri-menopausal and post-menopausal stages, rather than pure pre- versus post-menopausal differences. Nevertheless, this pattern of differences proved to be highly robust, as it remained consistent and significant even when we excluded women likely undergoing peri-menopause (based on menstrual cycle data) from the pre-menopausal group, and when we analyzed the youngest subgroup of post-menopausal women. Similarly, results remained unchanged in all our sensitivity analyses, in which we adjusted for several additional confounders (such as menarche age, use of oral contraceptives, body mass index, number of pregnancies, and adverse life events), or excluded women with a diagnosis of mental illness or those who had used menopausal hormone therapy.

Our main finding revealed that the post-menopausal group had larger gray matter volumes than the pre-menopausal group. This is in line with a recent study that also analyzed the UK Biobank (Ambikairajah et al. 2021), which reported larger total brain and hippocampal volumes in post-menopausal women. However, our result contrasts with previous research (Mosconi et al. 2021; Ramli et al. 2023). In fact, the majority of the studies in the field report smaller volumes in fronto-temporal regions of the brain, including the hippocampus, in post-menopausal women compared to pre-menopausal women (Ramli et al. 2023). According to Ambikairajah et al. 2021, this inconsistency may be attributed to a lack of age matching between pre-menopausal and post-menopausal groups in other studies, as well as sample size limitations. Nevertheless, two other studies using the UK Biobank dataset also found reduced gray matter volumes in post-menopausal women (Than et al. 2021; Zuhlsdorff et al. 2026) (see Table 1), particularly in the hippocampus, the entorhinal cortex, and the anterior cingulate cortex (Zuhlsdorff et al. 2026). Discrepancies between studies using the same dataset may be explained by the use of different inclusion criteria, statistical approach, and/or the specific covariates included in the analyses. In the particular case of our study, there are major methodological differences compared to previous UK Biobank studies. First, previous studies relied on the image-derived phenotypes provided by UK Biobank, which were processed using FreeSurfer. Conversely, we performed our own processing of the raw MRI data using CAT12. Importantly, CAT12 and FreeSurfer are known to produce different estimates of brain structure parameters, particularly in the temporal lobe (Seiger et al. 2018). This pipeline choice introduces unquantified variance, making direct comparisons with previous studies methodologically challenging. Furthermore, we employed whole-brain morphometry techniques (VBM and SBM), in contrast to previous UK Biobank studies, which had either performed region-of-interest (ROI) analyses or reported differences in global gray matter volumes. For instance, Than et al. 2021 reported differences in total gray matter volume, Ambikairajah et al. 2021 assessed hippocampal volume and total gray matter volume, and Zuhlsdorff et al. 2026 assessed only hippocampal, entorhinal, and anterior cingulate volumes. While ROI approaches offer well-established advantages in terms of statistical power and theoretical grounding, our whole-brain VBM and SBM approach provides an alternative, more exploratory perspective. It allows for a more precise spatial assessment of brain structure patterns across the entire brain, without a priori regional restrictions, offering unique insights that extend and complement previous global or ROI-based findings.

The characteristics of our sample warrant careful consideration. Given our reliance on self-reported data and the absence of menopausal staging questionnaires, such as the Stages of Reproductive Aging Workshop + 10 (STRAW + 10) criteria (Harlow et al. 2012), it is plausible that peri-menopausal women were inadvertently included in our pre-menopausal group. Previous research has reported lower gray matter volume in some regions in peri-menopausal women as compared with post-menopausal women, as well as gray matter volume increases in post-menopausal women after a 2-year follow-up, which could be explained by compensatory mechanisms (Mosconi et al. 2021). Hence, it is possible that our results are capturing the contrast between a hypothesized peri-menopausal trough and a subsequent post-menopausal recovery—although this is yet to be proven with further longitudinal studies—rather than a true difference between strictly pre-menopausal and post-menopausal women. By restricting our inclusion criteria to participants under 58 years old, we attained a relatively young post-menopausal group (mean age of 55 years). In contrast, other UK Biobank studies analyzed much older post-menopausal groups (62 years old and higher) (Ambikairajah et al. 2021; Than et al. 2021; Zuhlsdorff et al. 2026). If the structural differences observed in our sample reflect transient compensatory mechanisms (Mosconi et al. 2021), it is logically more probable for these older groups to have already surpassed the hypothetical window where these mechanisms would take place than for our younger post-menopausal group. Interestingly, Ambikairajah et al. 2021 still reported higher total gray matter and hippocampal volumes in post-menopausal women even after analyzing age-matched groups. Another caveat of having a much older post-menopausal than pre-menopausal group is that it is challenging to properly model the effects of age—and particularly its potential non-linear effects (Hedman et al. 2011)—on the brain. Although our groups were not strictly age-matched, our sensitivity analysis employing only the youngest post-menopausal women—which reduced the age effect size from very large (Cohen’s d = 1.57) to moderate (Cohen’s d = 0.49)—yielded a virtually identical pattern of results. However, it is still unclear why Ambikairajah et al. 2021 and the other UK Biobank studies report different results, and more research in this direction is needed.

To the best of our knowledge, this is the first study to apply whole-brain VBM and SBM techniques to assess the associations between menopausal status and both cortical thickness and cortical gyrification. While we did not find significant gyrification differences between groups, we observed greater cortical thickness in post-menopausal women, predominantly in the frontal lobe and the supramarginal gyrus. Altogether, our gray matter volume and cortical thickness findings point to differences in brain regions that are critically involved in Alzheimer’s disease; specifically, post-menopausal women showed larger gray matter volumes in the medial temporal lobe (including the hippocampus, the parahippocampal gyrus, and the amygdala) (Yang et al. 2012; Núñez et al. 2020), and greater cortical thickness in the supramarginal gyrus, a region where cortical thinning has been implicated in the early stages of Alzheimer’s disease (Dickerson et al. 2009). In fact, according to the Neurosynth Image Decoder tool, the functional and cognitive terms most closely associated with our anatomical findings are those related to memory and emotion. It is also interesting to note that estradiol receptors are abundant in most of the regions where we found prominent differences between groups, particularly the hippocampus (Österlund and Hurd 2001; González et al. 2007; Mosconi et al. 2024). This suggests that brain changes associated with menopause may be driven by estrogens or a change in the estrogen receptor landscape. Indeed, estrogen receptor alpha appears to be up-regulated after menopause in some of these regions, albeit more prominently in the middle frontal gyrus, perhaps providing further support to the compensatory hypothesis (Mosconi et al. 2024). It is unclear whether (and how) these hypothetical compensatory mechanisms are associated with the risk of developing Alzheimer’s disease later in life. Interestingly, a biphasic model for brain structural changes in Alzheimer’s disease has been proposed, by which cortical thickening seems to precede neurodegeneration (Pegueroles et al. 2017; Montal et al. 2021). Speculatively, this compensatory strategy in post-menopausal women could be associated with a heightened neuroinflammatory state, which might contribute to an increased vulnerability to neurodegenerative processes (Zhang et al. 2023). It is important to emphasize that this interpretation remains speculative, as the biphasic model of Alzheimer’s may not be applicable to healthy populations like the one employed in the present study. Furthermore, the associations derived from the Neurosynth Image Decoder must be interpreted conservatively, as this tool is primarily designed for meta-analytic functional, rather than structural, inference. In any case, our results suggest that menopause represents a period of critical brain changes with potentially important clinical consequences. These unresolved questions, along with the increased burden of neurological disorders in women and rapid population ageing, warrant further research with longitudinal data and older cohorts to shed light on the relationship between menopause and the onset and progression of psychiatric disorders, dementias, and cognitive impairment (Briceno Silva et al. 2024).

A striking observation in our study is the complete anatomical dissociation between the regions showing larger gray matter volume and those exhibiting greater cortical thickness in post-menopausal women. From a mechanistic perspective, this lack of spatial overlap may reflect distinct biological processes associated with menopause across different brain regions. Such dissociation aligns with the distinct nature of these morphometric indices. Although gray matter volume is a composite measure influenced by both cortical thickness and surface area (Winkler et al. 2010), volumetric differences can manifest completely independently of cortical thickness variations, pointing to distinct localized tissue alterations. For example, volumetric differences might be associated with dendritic arborization or oedema, while cortical thickness differences could be related to synaptic density or myelination. Regarding the absence of significant differences in cortical gyrification, this null finding is perhaps less surprising. Cortical folding is essentially a neurodevelopmental process; gyral and sulcal patterns are largely determined during gestation and shortly after birth, remaining relatively stable after this period (Armstrong et al. 1995). Therefore, it is biologically plausible that the menopausal transition, which occurs in midlife, is associated with structural variations in gray matter volume and cortical thickness without co-occurring differences in cortical folding—or at least not to an extent detectable with the techniques and statistical thresholds applied in our study.

To ensure the robustness and consistency of our results, we carried out several analyses adjusting for additional covariates or excluding a subset of participants. Since exposure to higher estrogen levels throughout the lifespan can have an impact on brain structure (Schelbaum et al. 2021; Ruehr et al. 2025; Mervosh and Devi 2025), we took into account relevant reproductive history variables, such as number of pregnancies and menarche age, and the original results did not change. We also reanalyzed the data after excluding women diagnosed with any mental illness and those who had used menopausal hormone therapy, as these factors can also affect brain structure (Ambikairajah et al. 2021; Mosconi et al. 2021). In particular, menopausal hormone therapy is intended to alleviate menopausal symptoms by administering hormones that naturally decline during the menopausal transition, although findings from randomized, placebo-controlled clinical trials remain inconsistent regarding its effects on brain structure (Coker et al. 2014; Albert et al. 2017; Kantarci et al. 2018, 2026). Notwithstanding these inconsistencies, our sensitivity analyses excluding these participants yielded the same spatial pattern as our original results, confirming that these findings were not driven by participants using menopausal hormone therapy. Additionally, we excluded women from the pre-menopausal group reporting irregular menstrual cycles (Harlow 2018), since they were likely undergoing peri-menopause. Gray matter volume and cortical thickness differences between this smaller, stricter pre-menopausal group and post-menopausal women remained almost identical to our original findings. In contrast, the comparison between post- and peri-menopausal women yielded a more spatially restricted pattern that only partially overlapped with our main results, while the comparison between strictly pre- and peri-menopausal women yielded no significant differences. Although the observed pattern might theoretically argue against the compensatory hypothesis, we cannot rule it out given the limitations of these comparisons. Specifically, these exploratory findings must be interpreted with extreme caution, as the analyses involving these smaller subgroups are likely underpowered. Additionally, we did not exclude participants based on endocrine or other conditions that could have led to irregular cycles, thereby potentially introducing heterogeneity into the peri-menopausal subgroup. These limitations, along with the fact that the post-menopausal group showed greater gray matter volume and cortical thickness than both the pre- and peri-menopausal groups, suggest that these two groups may be phenotypically mixed. Therefore, further research with well-defined pre- and peri-menopausal groups is needed to confirm these preliminary findings. Finally, to ensure that aging was not driving our results—despite controlling for age in our analyses—we examined a subgroup of males in the same age range as our female sample. As expected, older age was strictly associated with lower gray matter volume and cortical thickness throughout the brain. Although the peak effect sizes of these analyses (maximum Cohen’s d of 0.27 for gray matter volume and 0.28 for cortical thickness) are comparable in magnitude to those observed in the comparison between pre- and post-menopausal women (maximum Cohen’s d of 0.23 for gray matter volume and 0.19 for cortical thickness), the spatial pattern of results was globally widespread in association with age, but more regionally restricted in relation to menopause. While this contrasts with our menopause-related findings and could further support that the observed structural differences are specific to menopause, these results must also be interpreted cautiously, as a potential age-by-sex interaction unrelated to menopause cannot be entirely ruled out.

It is worth noting that the analysis of large datasets, such as the UK Biobank or the Adolescent Brain Cognitive Development study, enables the identification of subtle but potentially meaningful effects (Smith and Nichols 2018; Dick et al. 2021). Even though the effect sizes of our findings are small, with a maximum Cohen’s d value of 0.23, we believe they are biologically and clinically relevant. We estimated a mean gray matter volume difference of 1.87% between pre- and post-menopausal women across the largest cluster showing significant group differences. For reference, this difference is directly comparable to the estimated gray matter loss over a 2-year period in the hippocampus (1.76%) and the amygdala (2.02%) in a sample of healthy elderly participants (Fjell et al. 2009). Given the magnitude of the menopause-related structural differences described in this study, it is unlikely that these findings will have practical utility at the individual level for guiding personalized treatments or taking preventive actions. However, these subtle, population-level differences are still highly valuable, as they advance our understanding of the neurobiological mechanisms underlying menopause and shed light on how the menopausal transition may alter normative aging trajectories, potentially predisposing women to future cognitive decline and more severe clinical conditions.

### Strengths and limitations

The present study has several strengths. To the best of our knowledge, this is the first study to apply whole-brain VBM and SBM techniques to assess the associations between menopausal status and both cortical thickness and cortical gyrification. Furthermore, although previous studies on this topic have been published using the UK Biobank dataset, we employed a whole-brain approach with VBM and SBM techniques that allows for a more precise spatial assessment of differences throughout the brain without pre-specifying regions of interest. Finally, we controlled our analyses for several potential confounders to ensure the robustness of our results. In terms of limitations, the fact that this is a cross‐sectional study limits the potential for etiological conclusions. Consequently, our design precludes us from adjudicating between the different mechanistic interpretations proposed to explain our findings. However, we view these interpretations as potentially complementary and interrelated, rather than mutually exclusive competing frameworks. Also, although collected in the UK Biobank, information on the age at menopause onset was unavailable in our dataset, and the main variable assessing menopause status did not ask for a specific timeframe. Furthermore, our menopausal status classification did not rely on the established STRAW + 10 staging system (Harlow et al. 2012). Since menstrual bleeding patterns and endocrine biomarkers (e.g., follicle-stimulating hormone, anti-Müllerian hormone) are not collected in sufficient detail or are unavailable in the UK Biobank, the exact biological boundaries between reproductive stages could not be reliably determined. Collectively, this could have led to the misclassification of peri-menopausal women into either our pre- or post-menopausal group. Considering the post-menopausal structural recovery hypothesis reported in previous research (Mosconi et al. 2021), if peri-menopausal women were inadvertently included in the post-menopausal group, this would likely have attenuated the observed differences. Conversely, the inclusion of peri-menopausal women in the pre-menopausal group could have also confounded our comparisons. We partially mitigated this concern using menstrual cycle length data (Harlow 2018) to identify a subgroup of women likely undergoing peri-menopause. The sensitivity analysis excluding this subgroup yielded almost identical results. Additionally, since we did not exclude women taking medications that could impact menstrual bleeding patterns (e.g., oral contraceptives, intrauterine devices, estrogen patches, or chemotherapy) or with pre-existing endocrine conditions that may affect cycle length and regularity (e.g., polyendocrine metabolic ovarian syndrome or thyroid disorders), some women in reproductive stages could have been misclassified as peri- or post-menopausal. A related major limitation, inherent to the nature of the UK Biobank dataset, is that we lacked a younger group of women (e.g., aged 35 years or less) to serve as a pure pre-menopausal reference, as the youngest participants included in this group were 45 years old. Without such a reference, we cannot determine whether the larger gray matter volumes and greater cortical thickness observed in post-menopausal women represent a genuine structural gain, a failure or delay in the expected age-related decline, or a residual age confound. Moreover, the absence of biological hormonal measures, such as follicle-stimulating hormone, anti-Müllerian hormone, or estradiol, means that the estrogen-driven mechanisms proposed to explain our findings cannot be directly tested with the current data. In addition, the unbalanced group sizes in our main analysis could have inflated Type I errors towards the larger group. We addressed this by configuring the model to assume unequal variances, reducing the risk of false positives; moreover, we carried out a sensitivity analysis with balanced groups, which yielded a very similar spatial pattern of brain differences, confirming the robustness of our results. Finally, we acknowledge that the exclusion of non-white participants to minimize sample heterogeneity restricts the generalizability of our results to white European-ancestry populations. This is important given the known racial and ethnic differences in dementia prevalence (Mehta and Yeo 2017) and menopause symptom severity (Kochersberger et al. 2024).

## Conclusion

Compared to pre-menopausal women, post-menopausal women showed larger gray matter volumes in the medial temporal lobe and adjacent temporo-occipital regions, as well as greater cortical thickness in the frontal lobe and the supramarginal gyrus. These structural differences may reflect compensatory mechanisms or underlying inflammatory processes. Given the anatomical overlap between these regions and those affected in the early stages of Alzheimer’s disease, further research is necessary to elucidate how brain changes associated with menopause influence the risk of dementia.

## Supplementary Information

Below is the link to the electronic supplementary material.

Supplementary file1 (PDF 2976 KB)

## Data Availability

We used a specific UK Biobank dataset with project ID: 72377.
