# Supplementary material for: Greater temporo-occipital gray matter volume and frontoparietal cortical thickness in post-menopausal women: a UK Biobank study
Source: Brain Struct Funct. 2026 Oct 9;231(8):151. doi: 10.1007/s00429-026-03206-5 (PMC13652777; doi:10.1007/s00429-026-03206-5)

Greater temporo-occipital gray matter volume and frontoparietal cortical thickness in post-menopausal women: a UK Biobank study

Stefanos Tyrovolas, Maria J. Portella, Judith Usall, Maria Rosa Cambra-Martí, Antonio Callén, Josep Maria Haro, Christian Núñez, Christian Stephan-Otto

Supplementary Material

Supplementary Table 1. List of additional covariates and exclusion criteria tested in the voxel- and surface-brain morphometry sensitivity analyses.

Variable name	UK Biobank data-field(s)	Description
**Additional covariates**		
Menarche age	2714-2.0	Age when periods started
Ever used oral contraceptives	2784-2.0	Ever taken oral contraceptive pill
Years using oral contraceptives	2794-2.0 and 2804-2.0	Difference between data-field 2804-2.0 (age when last used oral contraceptive pill) and 2794-2.0 (age started oral contraceptive pill)
Number of live births	2734-2.0	
Number of total pregnancies	2734-2.0, 3829-2.0, 3839-2.0, 3849-2.0	Sum of the 4 data-fields (number of live births, number of stillbirths, number of spontaneous miscarriages, and number of pregnancy terminations)
Body mass index	21001-2.0	
Sleep duration	1160-2.0	Hours of sleep in every 24 hours (including naps)
Physical activity	22037-2.0, 22038-2.0, and 22039-2.0	Sum of the 3 data-fields (Metabolic Equivalent Task [MET] minutes per week for walking, moderate activity, and vigorous activity)
Current tobacco use	1239-2.0	
Past tobacco use	1249-2.0	
Current alcohol use	1558-2.0	
Townsend deprivation index	189-0.0	Census-based index of material deprivation, calculated at recruitment
Experiences following childhood	20521-0.0, 20522-0.0, 20523-0.0, 20524-0.0, and 20525-0.0	Sum of the 5 data-fields from a mental health questionnaire regarding experiences after age 16. Items 20522-0.0 and 20525-0.0 were reversed-scored for consistency with the other data-fields
Serious life events	20526-0.0, 20527-0.0, 20528-0.0, 20529-0.0, 20530-0.0, and 20531-0.0	Total count of any “yes” response (regardless of timing) across the 6 data-fields from a mental health questionnaire regarding lifetime serious events
Ever depressed for a whole week	4598-2.0	Ever had a time of feeling depressed or down for at least a whole week
Overall quality MRI	-	Quality metric obtained after MRI processing
Euler number	-	Quality metric for cortical reconstruction obtained after MRI processing

Additionally excluded participants

Diagnosed with any mental illness	41270-0.x and 41280-0.x	Exclusion of participants with a diagnosis of any mental illness before MRI, including dementia, schizophrenia, bipolar/mania, depression, anxiety/phobia, among others
Menopausal hormone therapy	2814-2.0	Exclusion of participants who had used menopausal hormone therapy (reported at the imaging visit)

Supplementary Table 2. Comparison of sociodemographic, reproductive, and clinical variables between pre-menopausal and the youngest post-menopausal women. Values are expressed as mean ± SD for continuous variables or percentages for categorical variables.

Variable	Pre-menopausal women (n = 1,033)	Youngest post-menopausal women (n = 1,033)	Difference[e]
Age (years)	51.5 ± 2.3	52.4 ± 1.4	**p < 0.001**
Menarche age (years)	13.2 ± 1.6	13.1 ± 1.5	p = 0.574
Ever used oral contraceptives (% yes)	88.3	89.5	p = 0.370
Age started oral contraceptives	19.3 ± 4.3	19.1 ± 3.6	p = 0.286
Years using oral contraceptives	12.0 ± 9.7	11.6 ± 9.3	p = 0.431
Number of live births	1.5 ± 1.2	1.4 ± 1.2	**p = 0.018**
Number of total pregnancies	2.0 ± 1.6	1.9 ± 1.6	p = 0.070
Body mass index	26.2 ± 5.0	25.7 ± 4.7	**p = 0.016**
Sleep duration (h)	7.2 ± 1.0	7.0 ± 1.0	**p < 0.001**
Physical activity	2,536 ± 2,270	2,632 ± 2,379	p = 0.370
Current tobacco use (%)[a]	96 / 2 / 2	95 / 2 / 3	p = 0.770
Past tobacco use (%)[b]	53 / 18 / 14 / 14	49 / 20 / 13 / 19	**p = 0.033**
Current alcohol use (%)[c]	5 / 11 / 17 / 31 / 24 / 11	6 / 13 / 16 / 31 / 25 / 10	p = 0.896
Townsend deprivation index	-1.5 ± 2.8	-1.4 ± 3.0	p = 0.306
Experiences following childhood[d]	2 (4)	2 (4)	p = 0.126
Serious life events[d]	0 (1)	0 (1)	p = 0.145
Ever depressed for a whole week (% yes)	59.4	61.0	p = 0.463
Diagnosed with any mental illness (% yes)	3.6	4.1	p = 0.566
Menopausal hormone therapy (% yes)	8.0	24.8	**p < 0.001**

[a] Categories for current tobacco use: 0 = No; 1 = Only occasionally; 2 = Yes, on most or all days.

[b] Categories for past tobacco use: 0 = I have never smoked; 1 = Just tried once or twice; 2 = Smoked occasionally; 3 = Smoked on most or all days. Note that percentages do not add up to 100% due to rounding.

[c] Categories for current alcohol use: 0 = Never; 1 = Special occasions only; 2 = One to three times a month; 3 = Once or twice a week; 4 = Three or four times a week; 5 = Daily or almost daily. Note that percentages do not add up to 100% due to rounding.

[d] Reported as median (interquartile range)

[e] p values are derived from t-tests for continuous variables and chi-squared tests for categorical variables.

Supplementary Table 3. Brain regions showing greater gray matter volume in the youngest post-menopausal women (n = 1,033) compared to pre-menopausal women (n = 1,033), controlling for the 23 covariates described in the main text. Only the clusters that survived Bonferroni correction, accounting for the three brain parameters analyzed ($p < 0.0167$), are listed. For each cluster, we list the main regions in two columns: sorted by the % of total cluster volume localized within that region (% of cluster overlap) and sorted by the % of the total volume of the region covered by the cluster (% of region involvement). See Supplementary Figure 1A for a graphical representation of these differences.

Cluster size (p value)	Peak t value (Cohen's d)	MNI coordinates	% of cluster overlap	% of region involvement
7,643 (8.9×10^{-8})	5.6 (0.25)	35, -15, 2	Right cerebellum exterior (21%)	Cerebellar vermal lobules VIII-X (67%)
			Left cerebellum exterior (12%)	Right amygdala (51%)
			Cerebellar vermal lobules VIII-X (10%)	Right parahippocampal gyrus (32%)
			Right hippocampus (5%)	Right posterior insula (30%)
			Cerebellar vermal lobules I-V (5%)	Right planum polare (28%)
			Right parahippocampal gyrus (5%)	Right hippocampus (26%)
			Right fusiform gyrus (4%)	Cerebellar vermal lobules I-V (19%)
			Right lingual gyrus (4%)	Cerebellar vermal lobules VI-VII (19%)
			Right posterior insula (4%)	Right basal forebrain (12%)
			Right planum polare (3%)	Right fusiform gyrus (10%)
4,094 (3.5×10^{-5})	5.5 (0.24)	8, -86, 18	Right cuneus (19%)	Right cuneus (37%)
			Left cuneus (16%)	Left cuneus (32%)
			Right lingual gyrus (13%)	Right calcarine cortex (22%)
			Left lingual gyrus (9%)	Right occipital fusiform gyrus (20%)
			Right calcarine cortex (8%)	Right lingual gyrus (15%)
			Right occipital fusiform gyrus (8%)	Left calcarine cortex (13%)
			Left calcarine cortex (5%)	Left lingual gyrus (11%)
			Left occipital fusiform gyrus (3%)	Left occipital fusiform gyrus (8%)
			Right inferior occipital gyrus (2%)	Right inferior occipital gyrus (3%)

Cluster size (p value)	Peak t value (Cohen's d)	MNI coordinates	% of cluster overlap	% of region involvement
			Left inferior occipital gyrus (2%)	Left inferior occipital gyrus (2%)
3,706 ($7.3x10^{-5}$)	5.5 (0.24)	57, -12, -23	Right middle temporal gyrus (45%)	Right middle temporal gyrus (26%)
			Right inferior temporal gyrus (34%)	Right inferior temporal gyrus (25%)
			Right superior temporal gyrus (5%)	Right superior temporal gyrus (6%)
			Right inferior occipital gyrus (3%)	Right inferior occipital gyrus (4%)
			Right temporal pole (1%)	Right temporal pole (1%)
			Right fusiform gyrus (1%)	Right fusiform gyrus (1%)
1,444 (0.011)	4.7 (0.21)	-59, -35, -9	Left inferior temporal gyrus (53%)	Left inferior temporal gyrus (16%)
			Left middle temporal gyrus (36%)	Left middle temporal gyrus (8%)
			Left superior temporal gyrus (2%)	Left superior temporal gyrus (1%)
			Left fusiform gyrus (1%)	Left fusiform gyrus (1%)

Supplementary Table 4. Brain regions showing greater cortical thickness in the youngest post-menopausal women (n = 1,033) compared to pre-menopausal women (n = 1,033), controlling for the 23 covariates described in the main text. Only the clusters that survived Bonferroni correction, accounting for the three brain parameters analyzed ($p < 0.0167$), are listed. See Supplementary Figure 1B for a graphical representation of these differences.

Cluster size (p value)	Peak t value (Cohen's d)	MNI coordinates	Regions in cluster (%)
1,212 (1.4×10^{-5})	5.3 (0.23)	60, -27, 40	Right supramarginal (100%)
930 (2.4×10^{-4})	4.4 (0.19)	-53, -39, 51	Left supramarginal (100%)
823 (7.3×10^{-4})	3.6 (0.16)	34, 34, 27	Right rostral middle frontal (95%)
			Right superior frontal (5%)
758 (0.001)	4.2 (0.18)	7, -73, 41	Right superior parietal (61%)
			Right precuneus (39%)
743 (0.002)	5.1 (0.22)	-29, 42, 34	Left rostral middle frontal (65%)
			Left superior frontal (18%)
			Left caudal middle frontal (17%)
732 (0.002)	4.7 (0.21)	-30, 55, 13	Left rostral middle frontal (85%)
			Left superior frontal (15%)
687 (0.003)	3.9 (0.17)	14, 66, 9	Right rostral middle frontal (70%)
			Right superior frontal (30%)
636 (0.005)	4.0 (0.18)	-20, -60, 62	Left superior parietal (100%)
593 (0.007)	4.8 (0.21)	49, -39, 54	Right supramarginal (89%)
			Right inferior parietal (11%)

Supplementary Table 5. Brain regions showing greater gray matter volume in post-menopausal women (n = 3,065) compared to a subgroup of pre-menopausal women (n = 543) that excluded those likely undergoing peri-menopause, controlling for the 23 covariates described in the main text. Only the clusters that survived Bonferroni correction, accounting for the two brain parameters analyzed ($p < 0.025$), are listed. For each cluster, we list the main regions in two columns: sorted by the % of total cluster volume localized within that region (% of cluster overlap) and sorted by the % of the total volume of the region covered by the cluster (% of region involvement). See Supplementary Figure 3A for a graphical representation of these differences.

Cluster size (p value)	Peak t value (Cohen's d)	MNI coordinates	% of cluster overlap	% of region involvement
8,152 (2.4×10^{-8})	5.4 (0.25)	32, -15, -8	Right hippocampus (10%)	Right amygdala (99%)
			Left hippocampus (7%)	Left accumbens (73%)
			Right parahippocampal gyrus (5%)	Right hippocampus (58%)
			Right amygdala (5%)	Left amygdala (44%)
			Left temporal pole (4%)	Left hippocampus (42%)
			Right lingual gyrus (3%)	Right parahippocampal gyrus (36%)
			Left superior temporal gyrus (3%)	Right basal forebrain (21%)
			Left fusiform gyrus (2%)	Left basal forebrain (20%)
			Right posterior insula (2%)	Right posterior insula (18%)
			Right thalamus (2%)	Right entorhinal (17%)
7,357 (8.4×10^{-8})	5.4 (0.25)	9, -89, -2	Right inferior temporal gyrus (16%)	Right cuneus (40%)
			Right middle temporal gyrus (16%)	Right calcarine cortex (32%)
			Right cuneus (11%)	Right occipital fusiform gyrus (31%)
			Left cuneus (7%)	Left cuneus (25%)
			Right occipital fusiform gyrus (6%)	Right inferior temporal gyrus (24%)
			Right lingual gyrus (6%)	Right middle temporal gyrus (18%)
			Right calcarine cortex (6%)	Left calcarine cortex (16%)
			Right cerebellum exterior (4%)	Right lingual gyrus (14%)
			Left calcarine cortex (3%)	Right middle occipital gyrus (9%)

Cluster size (p value)	Peak t value (Cohen's d)	MNI coordinates	% of cluster overlap	% of region involvement
			Right middle occipital gyrus (3%)	Right superior occipital gyrus (9%)
1,863 (0.003)	5.4 (0.25)	-57, -33, -11	Left middle temporal gyrus (44%)	Left inferior temporal gyrus (14%)
			Left inferior temporal gyrus (36%)	Left middle temporal gyrus (13%)
			Left superior temporal gyrus (5%)	Left superior temporal gyrus (3%)
			Left fusiform gyrus (3%)	Left fusiform gyrus (2%)
			Left inferior occipital gyrus (2%)	Left inferior occipital gyrus (1%)
			Left occipital fusiform gyrus (1%)	Left occipital fusiform gyrus (1%)
1,286 (0.015)	4.9 (0.23)	-47, -63, 29	Left angular gyrus (84%)	Left angular gyrus (26%)
			Left superior parietal lobule (8%)	Left superior parietal lobule (2%)
			Left middle occipital gyrus (2%)	Left middle occipital gyrus (1%)

Supplementary Table 6. Brain regions showing greater cortical thickness in post-menopausal women (n = 3,065) compared to a subgroup of pre-menopausal women (n = 543) that excluded those likely undergoing peri-menopause, controlling for the 23 covariates described in the main text. Only the clusters that survived Bonferroni correction, accounting for the two brain parameters analyzed ($p < 0.025$), are listed. See Supplementary Figure 3B for a graphical representation of these differences.

Cluster size (p value)	Peak t value (Cohen's d)	MNI coordinates	Regions in cluster (%)
1,253 (6.6×10^{-6})	4.3 (0.20)	-52, -38, 51	Left supramarginal (100%)
930 (1.9×10^{-4})	4.9 (0.23)	-28, 54, 11	Left rostral middle frontal (92%)
			Left superior frontal (8%)
911 (2.4×10^{-4})	4.2 (0.20)	49, -11, 38	Right postcentral (100%)
815 (6.4×10^{-4})	4.8 (0.22)	-30, 40, 33	Left rostral middle frontal (51%)
			Left superior frontal (41%)
			Left caudal middle frontal (7%)
757 (0.001)	4.7 (0.22)	-2, -65, 15	Left cuneus (87%)
			Left pericalcarine (7%)
			Left precuneus (7%)
698 (0.002)	3.9 (0.18)	6, 67, -1	Right rostral middle frontal (47%)
			Right superior frontal (44%)
			Right frontal pole (10%)
600 (0.006)	4.5 (0.21)	24, 27, 53	Right superior frontal (100%)
474 (0.022)	3.9 (0.18)	19, -63, 61	Right superior parietal (100%)

Supplementary Table 7. Brain regions showing greater gray matter volume in post-menopausal women (n = 3,065) compared to a subgroup of women likely undergoing peri-menopause (n = 431), controlling for the 23 covariates described in the main text. Only the clusters that survived Bonferroni correction, accounting for the two brain parameters analyzed ($p < 0.025$), are listed. For each cluster, we list the main regions in two columns: sorted by the % of total cluster volume localized within that region (% of cluster overlap) and sorted by the % of the total volume of the region covered by the cluster (% of region involvement).

Cluster size (p value)	Peak t value (Cohen's d)	MNI coordinates	% of cluster overlap	% of region involvement
2,037 (0.002)	4.9 (0.25)	59, -32, -17	Right middle temporal gyrus (39%)	Right inferior occipital gyrus (14%)
			Right inferior temporal gyrus (27%)	Right middle temporal gyrus (12%)
			Right inferior occipital gyrus (18%)	Right inferior temporal gyrus (11%)
			Right occipital fusiform gyrus (4%)	Right occipital fusiform gyrus (6%)
			Right superior temporal gyrus (1%)	Right superior temporal gyrus (1%)

Supplementary Table 8. Brain regions showing greater cortical thickness in post-menopausal women (n = 3,065) compared to a subgroup of women likely undergoing peri-menopause (n = 431), controlling for the 23 covariates described in the main text. Only the clusters that survived Bonferroni correction, accounting for the two brain parameters analyzed ($p < 0.025$), are listed.

Cluster size (p value)	Peak t value (Cohen's d)	MNI coordinates	Regions in cluster (%)
633 (0.005)	4.1 (0.21)	28, 34, 37	Left rostral middle frontal (57%)
			Left superior frontal (35%)
			Left caudal middle frontal (8%)
545 (0.013)	4.4 (0.23)	37, 35, 29	Left rostral middle frontal (100%)

Supplementary Table 9. Top 100 terms identified by the Neurosynth Image Decoder tool for the unthresholded T-map corresponding to the contrast of interest from our original voxel-based morphometry analysis (showing larger gray matter volumes in the post-menopausal group).

#	Term	Correlation	#	Term	Correlation
1	hippocampus	0.210	51	inferior temporal	0.118
2	amygdala	0.193	52	lobe	0.118
3	medial temporal	0.192	53	valence	0.114
4	parahippocampal	0.184	54	extrastriate	0.113
5	hippocampal	0.181	55	ventral	0.113
6	temporal lobe	0.179	56	happy	0.113
7	anterior hippocampus	0.175	57	fearful faces	0.113
8	episodic	0.171	58	ventral visual	0.113
9	parahippocampal cortex	0.165	59	cortex amygdala	0.112
10	amygdala hippocampus	0.163	60	striatum	0.112
11	encoding	0.163	61	value	0.112
12	medial	0.163	62	reactivity	0.112
13	memories	0.160	63	perception	0.112
14	faces	0.160	64	fusiform gyrus	0.112
15	mtl	0.159	65	object	0.111
16	parahippocampal gyrus	0.159	66	amygdala insula	0.110
17	autobiographical	0.157	67	orbitofrontal cortex	0.110
18	occipito	0.155	68	arousal	0.110
19	emotional	0.152	69	monetary	0.110
20	lobe mtl	0.150	70	rewards	0.109
21	neutral	0.150	71	anxiety	0.109
22	face	0.149	72	emotional stimuli	0.109
23	temporal	0.147	73	aversive	0.109
24	episodic memory	0.146	74	navigation	0.109
25	pictures	0.145	75	recognition	0.108
26	accumbens	0.143	76	disgust	0.107
27	facial	0.143	77	motivation	0.107
28	occipito temporal	0.141	78	insula	0.107
29	fusiform	0.140	79	incentive	0.107
30	expressions	0.139	80	occipitotemporal	0.106
31	nucleus accumbens	0.139	81	angry	0.106
32	fearful	0.137	82	unpleasant	0.106
33	social	0.136	83	memory retrieval	0.106
34	objects	0.135	84	anticipation	0.104
35	default	0.133	85	fusiform face	0.104
36	fear	0.131	86	emotions	0.103
37	affective	0.130	87	retrosplenial	0.100
38	autobiographical memory	0.130	88	medial prefrontal	0.100
39	ventral striatum	0.129	89	orbitofrontal	0.099
40	retrieval	0.127	90	negative	0.099
41	facial expressions	0.125	91	occipital	0.099
42	default mode	0.124	92	memory	0.099
43	reward	0.123	93	limbic	0.099
44	amygdala response	0.123	94	temporal sulcus	0.098
45	neutral faces	0.122	95	amygdala anterior	0.098
46	viewing	0.122	96	visual stream	0.098
47	amygdala responses	0.121	97	temporal cortex	0.097
48	emotionally	0.120	98	reward anticipation	0.097
49	visual	0.119	99	motivational	0.097
50	memory encoding	0.118	100	posterior insula	0.095

Supplementary Figure 1. Histograms depicting the age distributions of pre- and post-menopausal women in the full sample (A) and the subsample including only the youngest post-menopausal women (B).

A

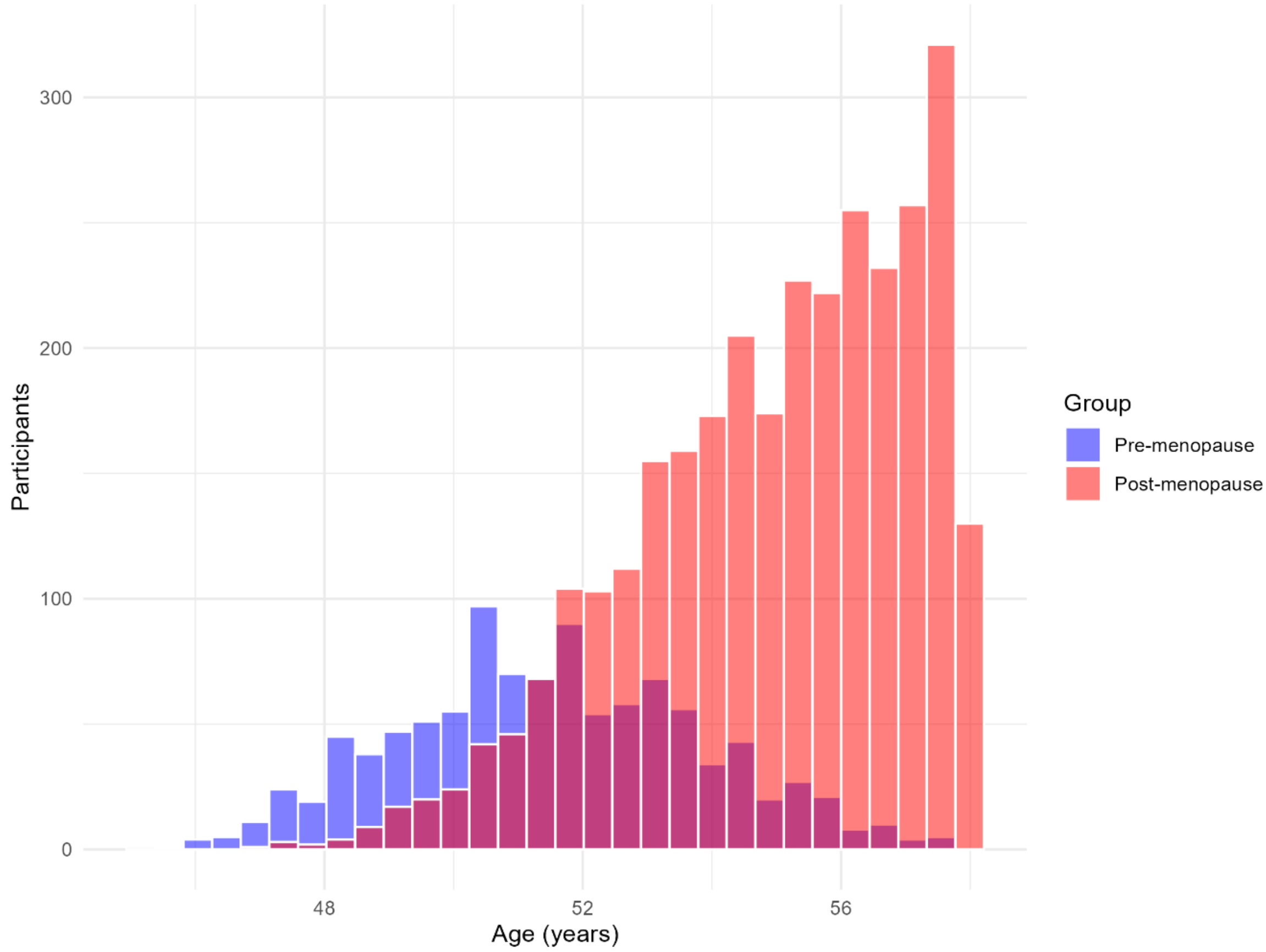

B

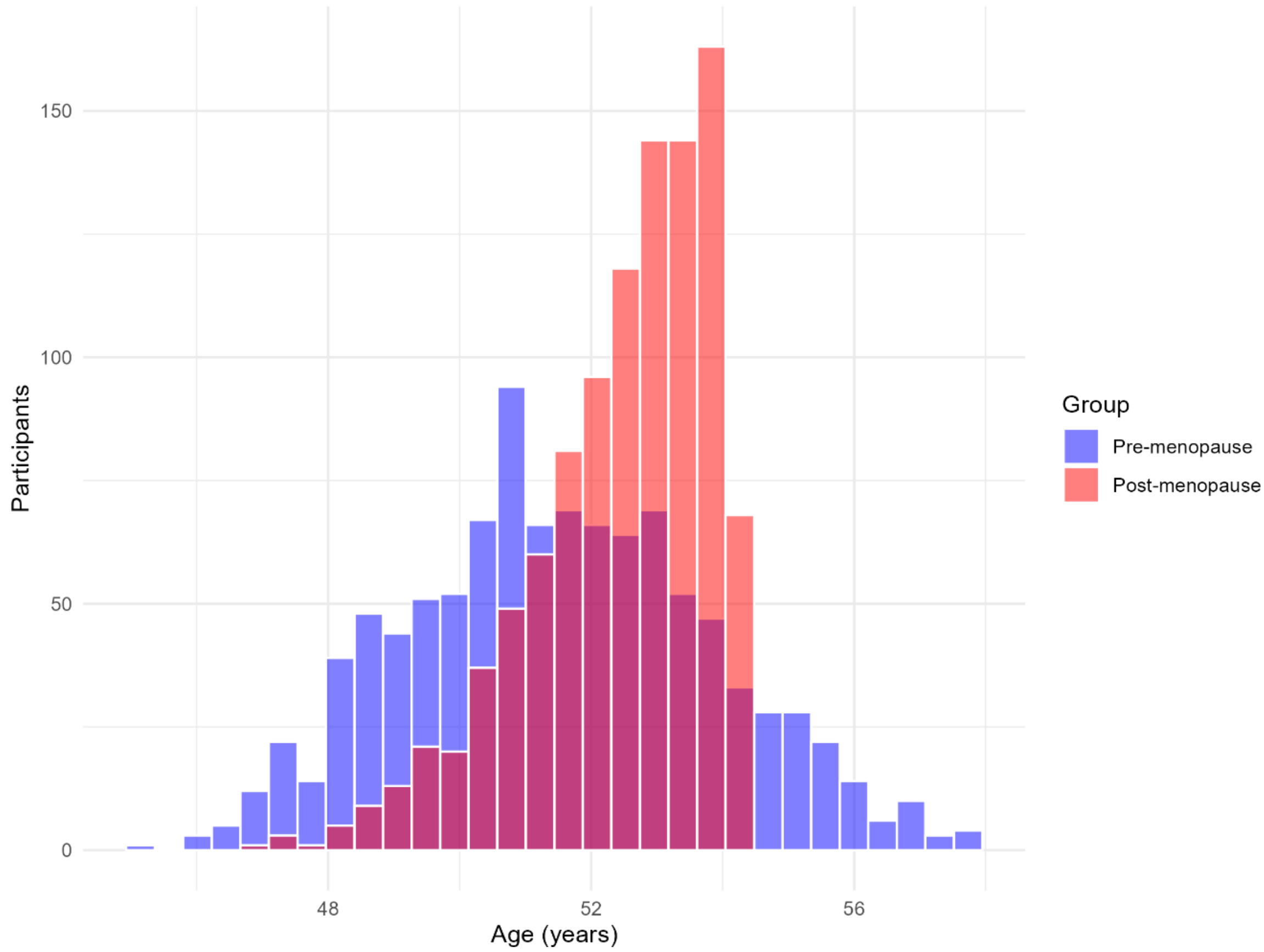

Supplementary Figure 2. Brain clusters where the youngest post-menopausal women (n = 1,033) showed greater gray matter volume **(A)** or cortical thickness **(B)** than pre-menopausal women (n = 1,033). Color bars represent T values and the numbers below the axial slices indicate the "z" MNI coordinate. See Supplementary Tables 2 and 3 for a list of the brain regions depicted in this figure.

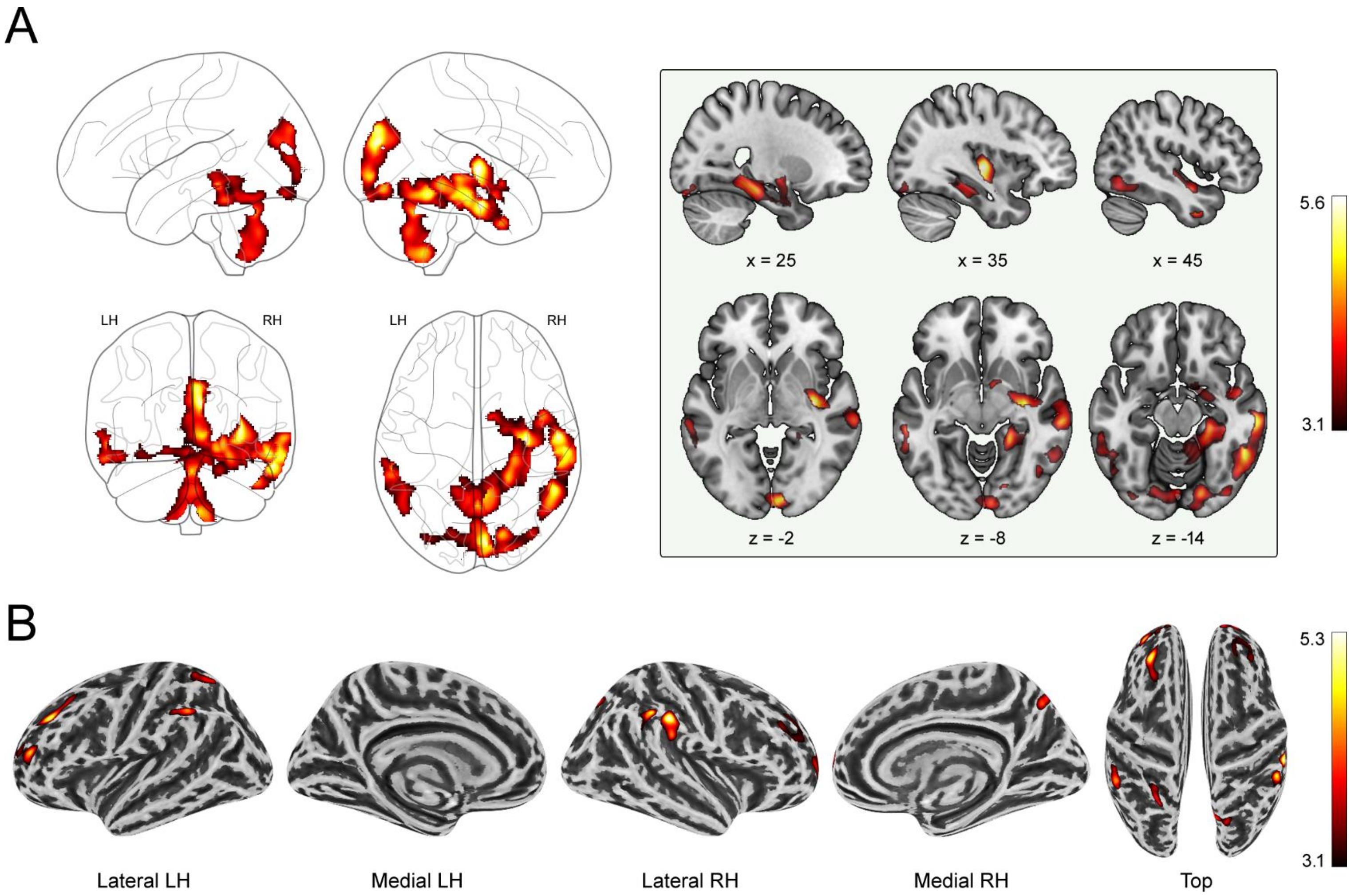

Supplementary Figure 3. Results of the whole-brain morphometry analyses for gray matter volume (left) and cortical thickness (right) after including additional covariates or after excluding women with a diagnosis of mental illness or who had used menopausal hormone therapy.

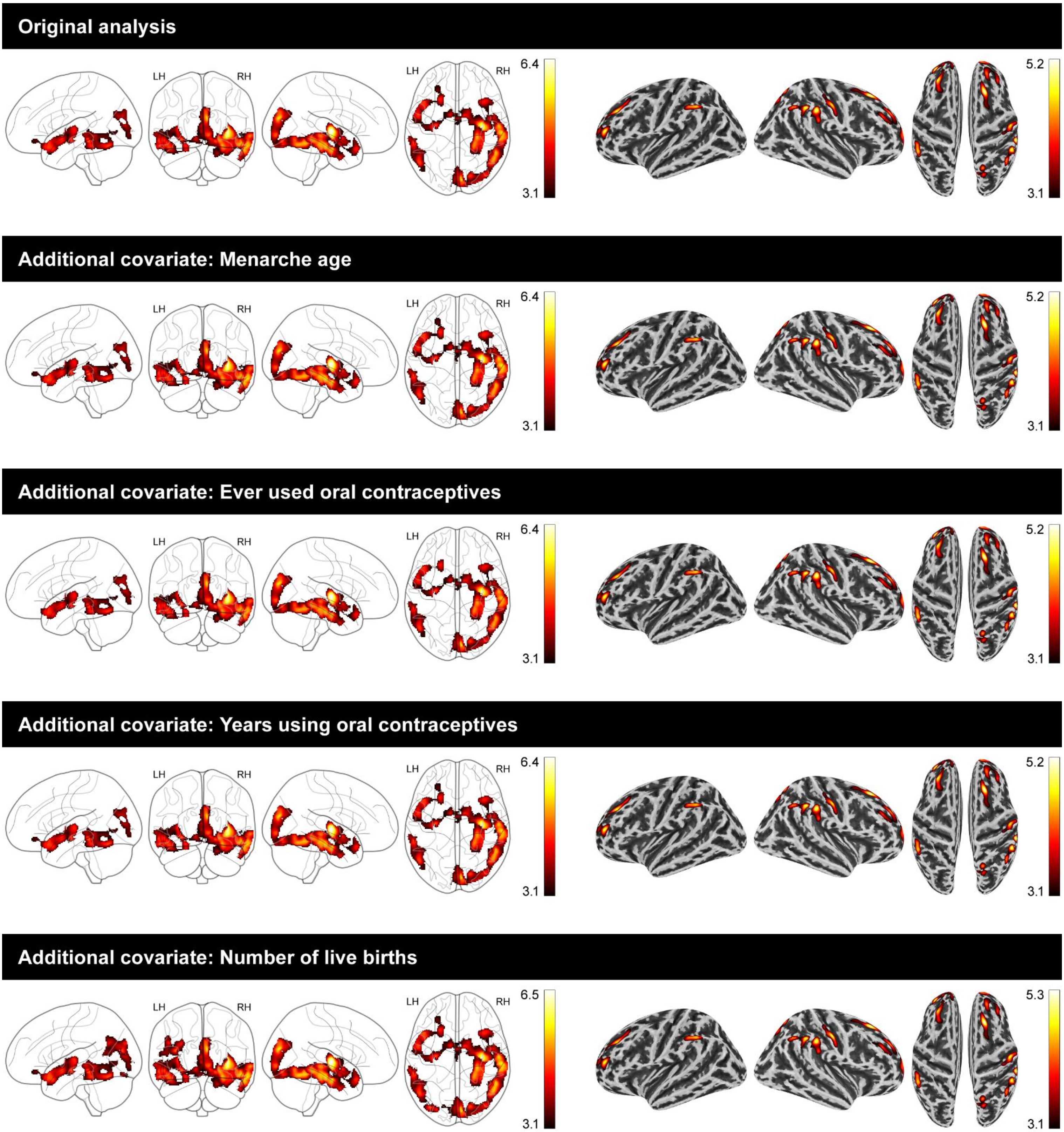

Supplementary Figure 3. (continuation)

Additional covariate: Number of total pregnancies

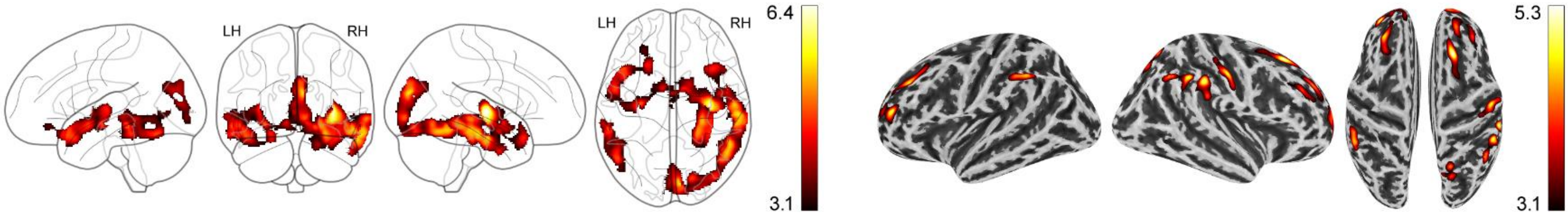

Additional covariate: Body mass index

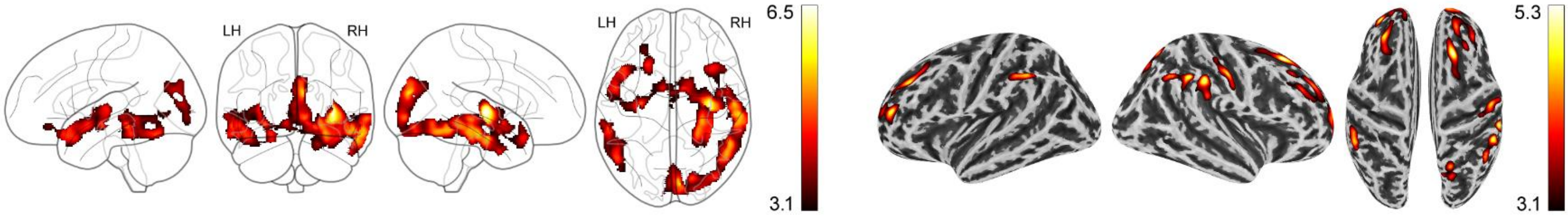

Additional covariate: Sleep duration

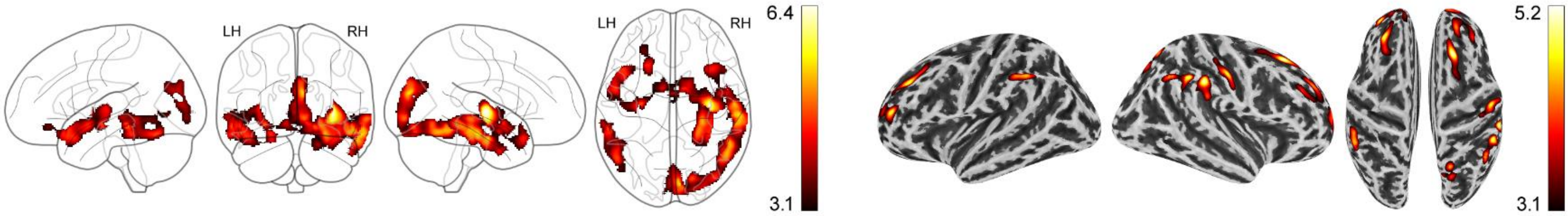

Additional covariate: Physical activity

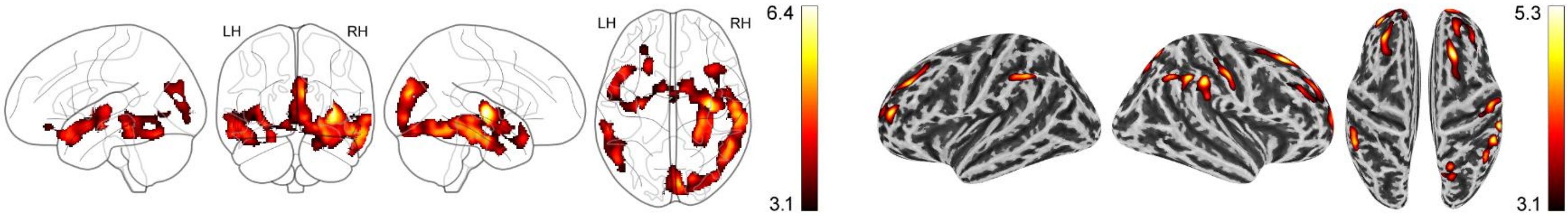

Additional covariate: Current tobacco use

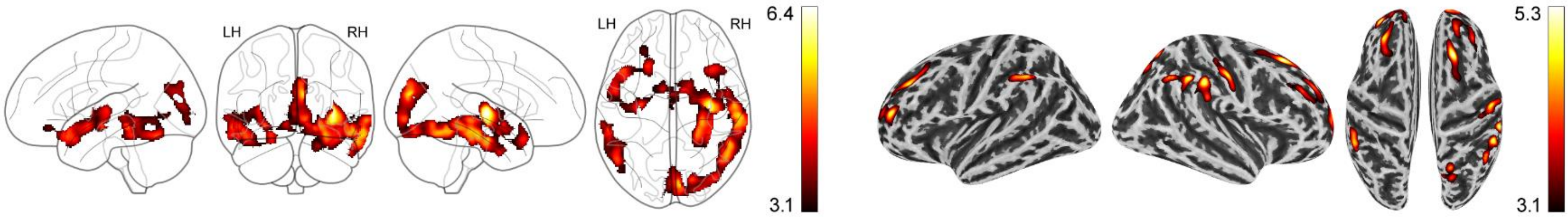

Supplementary Figure 3. (continuation)

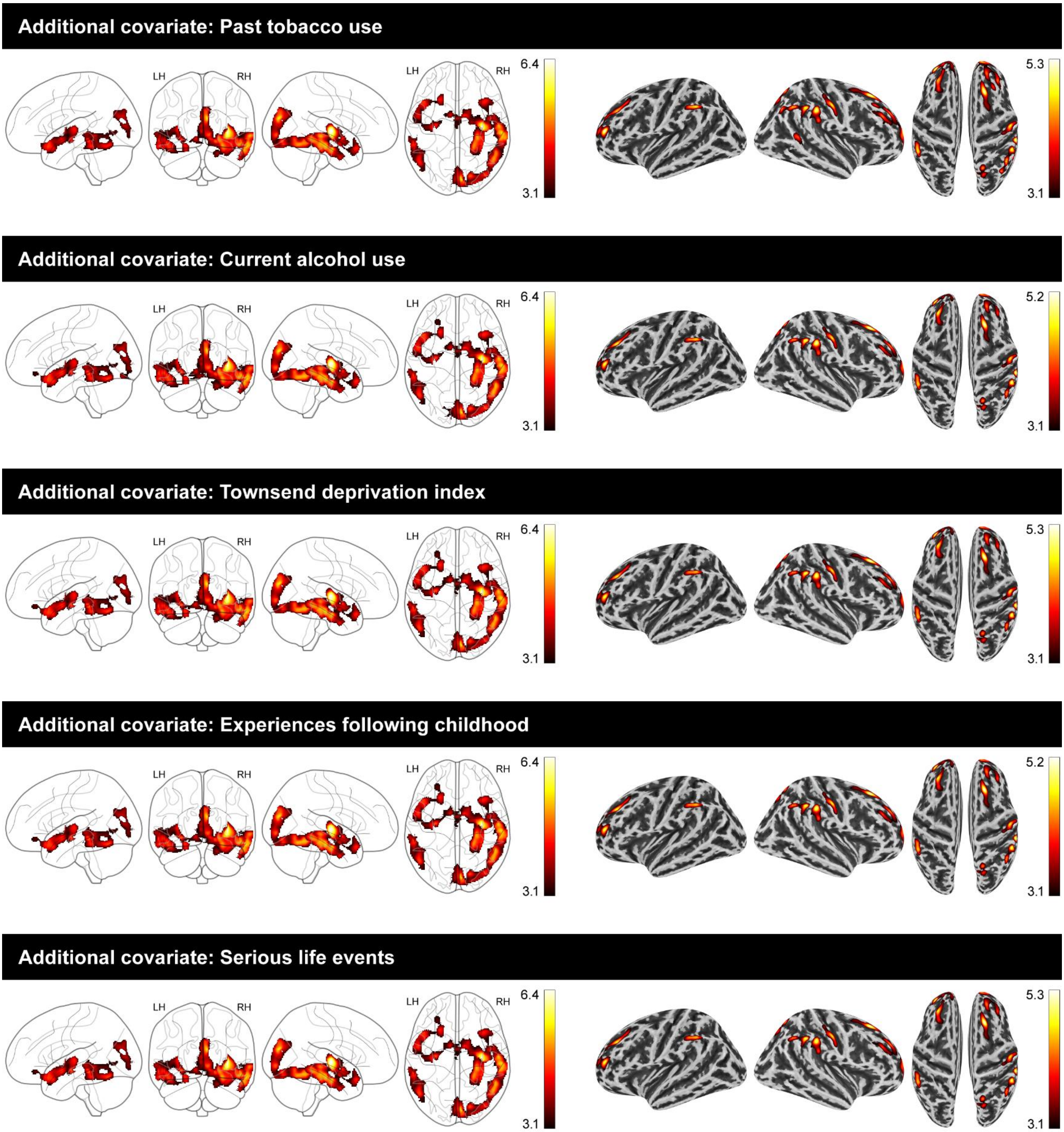

Supplementary Figure 3. (continuation)

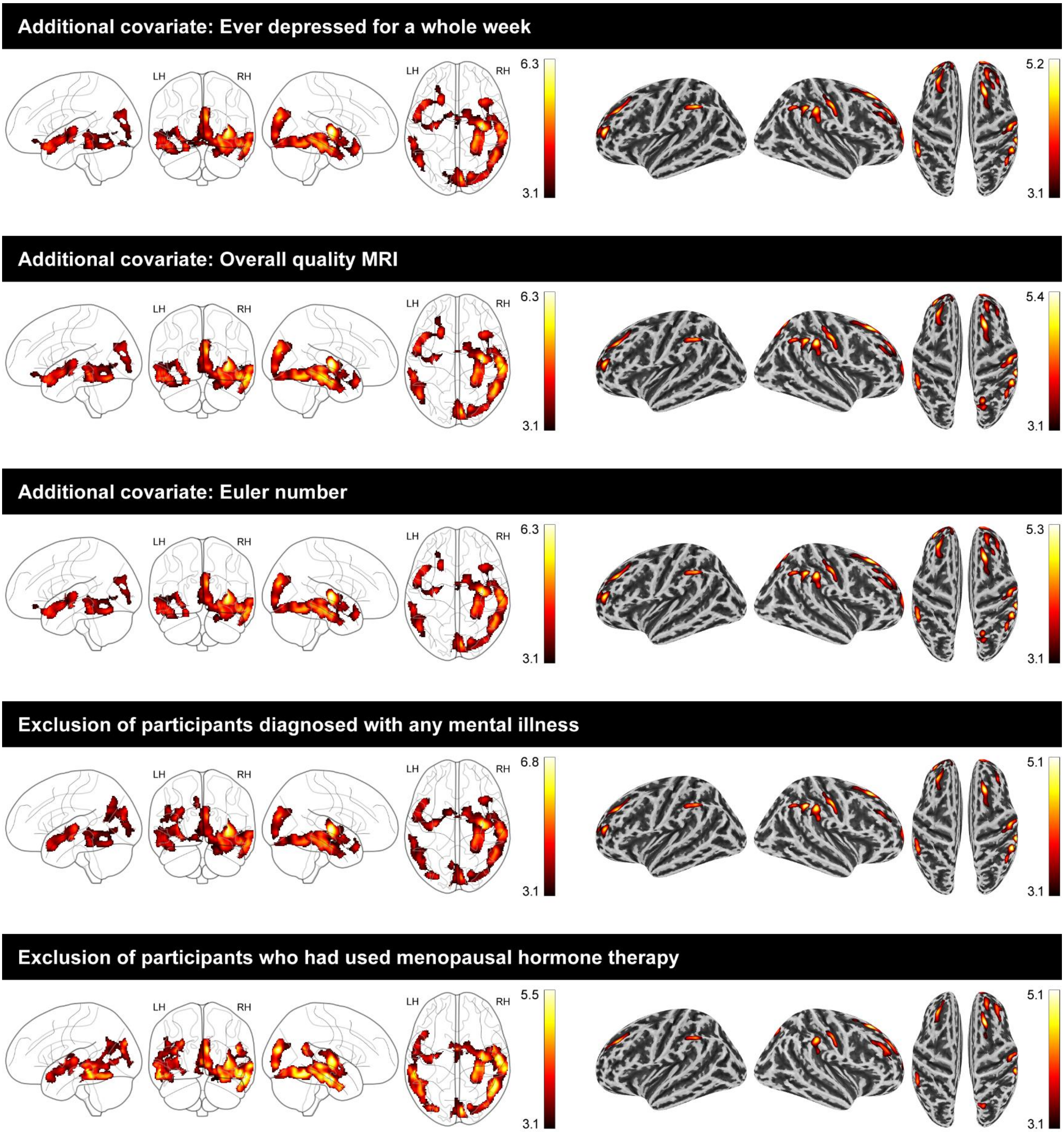

Supplementary Figure 4. Brain clusters where age was negatively associated with gray matter volume **(A)** or cortical thickness **(B)** in the male sample (n = 3,704). Color bars represent T values and the numbers below the axial slices indicate the "z" MNI coordinate.

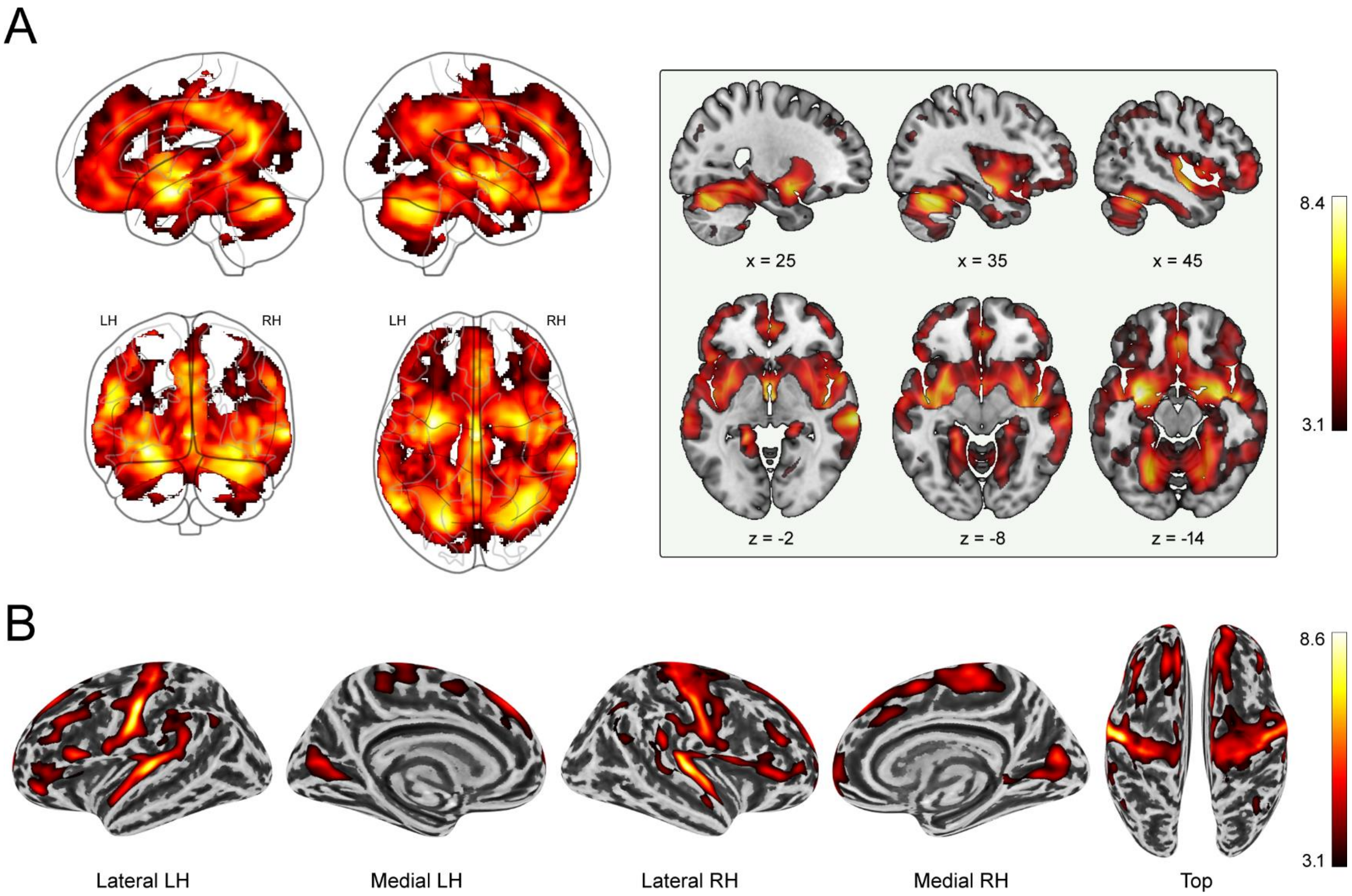

Supplementary Figure 5. Brain clusters where post-menopausal women (n = 3,065) showed greater gray matter volume **(A)** or cortical thickness **(B)** than a subgroup of pre-menopausal women (n = 543) that excluded those likely undergoing peri-menopause. Color bars represent T values and the numbers below the axial slices indicate the “z” MNI coordinate. See Supplementary Tables 4 and 5 for a list of the brain regions depicted in this figure.

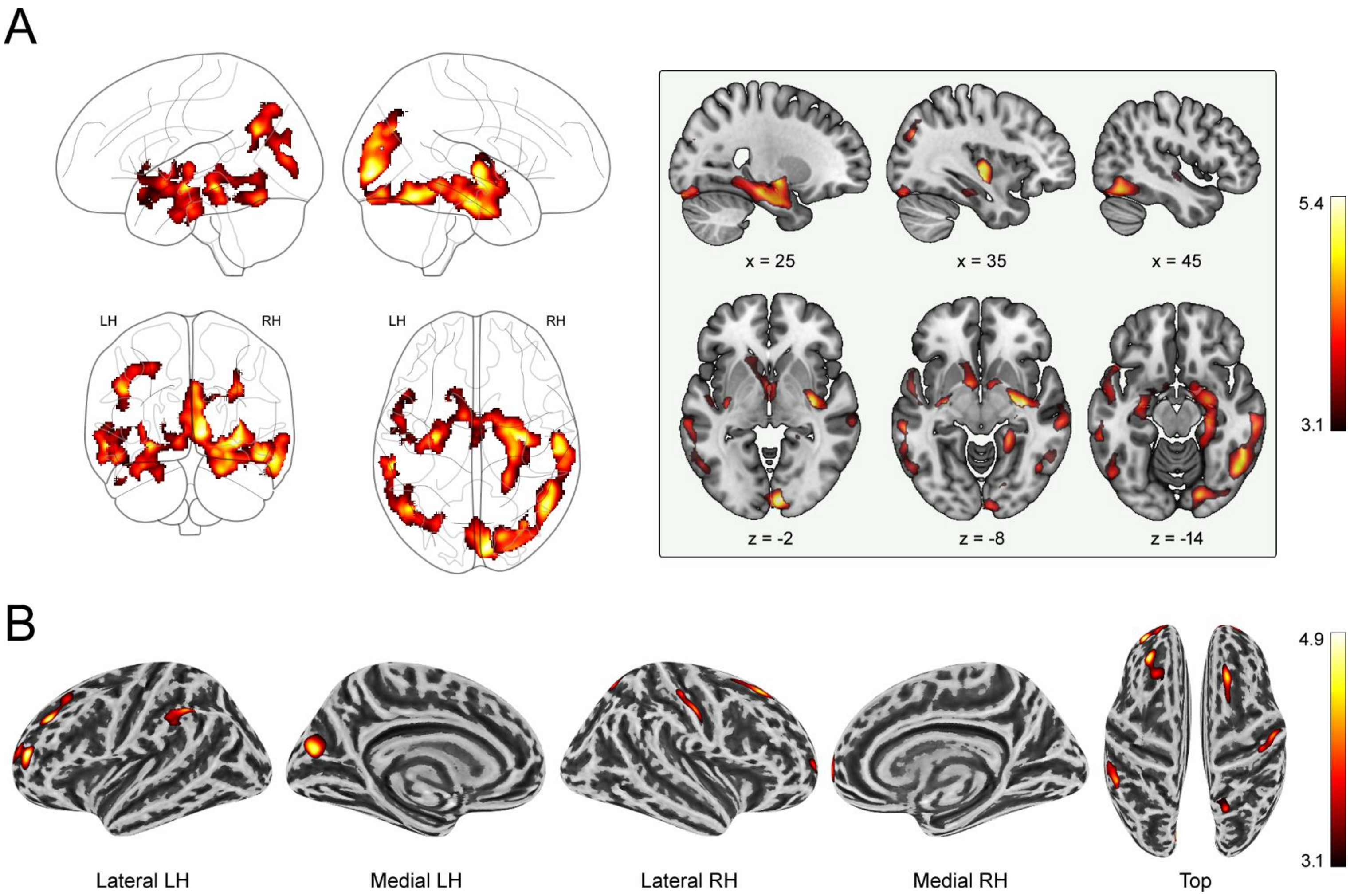

Supplement: Supplementary file 1 [file 429_2026_3206_MOESM1_ESM.pdf]
